# USP45 and Spindly are part of the same complex implicated in cell migration

**DOI:** 10.1038/s41598-018-32685-8

**Published:** 2018-09-26

**Authors:** Claudia Conte, Eric R. Griffis, Ian Hickson, Ana B. Perez-Oliva

**Affiliations:** 1 MRC Protein Phosphorylation and Ubiquitylation Unit, College of Life Sciences, University of Dundee, Dundee, UK; 2 Janssen Research & Development, LLC, Spring House, PA USA; 3 Present Address: Cell and Developmental Biology, Center of Genomic Regulation (CRG), Barcelona, Spain; 4 Present Address: Nikon Instruments Incorporated, Melville, NY 11747 USA; 5 Present Address: Northern Institute for Cancer Research, Newcastle University, Medical School, Newcastle upon Tyne, NE2 4HH UK

## Abstract

Ubiquitylation is a protein modification implicated in several cellular processes. This process is reversible by the action of deubiquinating enzymes (DUBs). USP45 is a ubiquitin specific protease about which little is known, aside from roles in DNA damage repair and differentiation of the vertebrate retina. Here, by using mass spectrometry we have identified Spindly as a new target of USP45. Our data show that Spindly and USP45 are part of the same complex and that their interaction specifically depends on the catalytic activity of USP45. In addition, we describe the type of ubiquitin chains associated with the complex that can be cleaved by USP45, with a preferential activity on K48 ubiquitin chain type and potentially K6. Here, we also show that Spindly is mono-ubiquitylated and this can be specifically removed by USP45 in its active form but not by the catalytic inactive form. Lastly, we identified a new role for USP45 in cell migration, similar to that which was recently described for Spindly.

## Introduction

Protein activity within the cell can be regulated through several post-translational modification processes to expand any given protein’s functional repertoire. Ubiquitylation is one of the major modifications that occurs in the cell; it consists of the addition of one or several subunits of the small and highly conserved molecule, called ubiquitin^1^. The addition of ubiquitin requires the action of three enzymes: the ubiquitin-activating enzyme (or E1), a step requiring ATP as energy source; the ubiquitin-conjugating enzyme (or E2), which allows the transfer of ubiquitin from E1 to E2 via a trans-(thio)- esterification reaction; and lastly the E3 ligase enzyme (or E3) that, by interacting with both the E2 and the substrate, generates an isopeptide bond between a lysine of the target protein and the C-terminal glycine of the ubiquitin molecule^2,3^. This process plays an important function in the regulation and in the degradation of many proteins depending on where and how many molecules of ubiquitin are attached to the target protein^4^. Ubiquitin molecules can be linked through one of the seven ubiquitin Lys residues (which are K6, K11, K27, K29, K33, K48 and K63) or through the ubiquitin amino terminal Met1 residue (which generates linear chains)^5^. Different roles have been associated with different kinds of chains^6^.

The ubiquitination process is reversible thanks to the activity of deubiquitinating enzymes (DUBs). So far, these enzymes have been divided into 5 families: Ubiquitin C-Terminal Hydrolases (UCHs), Ubiquitin-Specific Proteases (USPs), Machado-Joseph Disease Protein Domain Proteases (MJDs), Ovarian Tumor Proteases (OTUs), Monocyte Chemotactic Protein Induced Protein Proteases (MCPIP) and JAB/MPN/Mov34 Metalloenzyme (JAMM) domain Proteases^7,8^. Only the JAMMs are metallo proteases, while the rest of the families are cysteine proteases. The activity of DUBs has been implicated in several important pathways including cell growth and differentiation, development, oncogenesis, neuronal disease and transcriptional regulation. In addition, they have a crucial role in controlling proteasome dependent protein degradation, endocytosis and DNA damage repair^9–11^.

The largest family of DUBs is represented by USPs, papain-like isopeptidases with a common catalytic domain that varies greatly in size (50–300 kDa), and that usually contain an N-terminal domain with function in substrate recognition, subcellular localization and/or protein-protein interaction^12^. USPs can process ubiquitin precursors, remove ubiquitin from protein conjugates and disassemble ubiquitin chains. In general the members already characterized show a promiscuous activity against almost all ubiquitin chains. USP45 belongs to the USPs family of proteases and it has been shown to play a role in DNA damage regulating ERCC1 ubiquitin levels as well as its recruitment to DNA damage sites. It has been also described that USP45 can be recruited to DNA damage sites dependent upon its catalytic activity but independently of its interaction with ERCC-XPF complex^13^. On the other hand, a recent publication described a role of USP45 in vertebrate retina differentiation; using a morpholino approach the authors show that knockdown of USP45 showed a moderate to severe morphological eye defect in a zebrafish model. This phenotype is associated with retina formation and differentiation and the authors postulate USP45 as a candidate for causing hereditary retinal dystrophies^14^.

Spindly, also called CCDC99, is a coiled-coil domain containing protein, already described as an important regulator of the mitotic checkpoint^15–17^.

Spindly was identified through two RNAi screens in *Drosophila melanogaster* S2 cells, where its specific depletion generated alterations in cytoskeletal architecture and in mitotic progression^17^. After its discovery, several publications have been focused on describing the role of Spindly in mitosis^15,16,18–23^. Spindly plays an important role at kinetochores where it can interact with components of the RZZ complex (Rod/ZW10/Zwilch) as well as the dynein-dynactin complex^20,24,25^. In addition, a recent publication showed that Spindly associates with the cell cortex and colocalizes with dynein/dynactin at the leading edge of migrating cells. In this publication, the authors showed that cells lacking Spindly migrate slower than wild type cells and that this effect is rescued by re-expression of wild-type Spindly, whereas a mutant specifically defective in dynactin binding fails to rescue the phenotype^26^. These data are the first description of the importance of Spindly in the cell migration process in human cells.

In this study, we have used mass spectrometry to identify Spindly as a new interactor of USP45. Our data indicate that Spindly and USP45 are part of the same protein complex and that the interaction depends on the catalytic activity of USP45. In addition, we identified ubiquitin chains associated with the complex that can be cleaved by USP45, with a preferential activity on K48 ubiquitin chains. Moreover, we demonstrated that Spindly is not ubiquitylated in a K48-polyubiquitin manner, suggesting that there must be at least a third component of the complex. Interestingly, we could detect a monoubiquitylation of Spindly, which could be specifically removed by USP45. Additionally, in this study we show that USP45 plays a role analogous to Spindly in cell migration. Our data indicate that the lack of USP45 reduces migration velocity compared to wild-type cells.

## Results

### Spindly a new interactor of USP45

**Figure 1:**
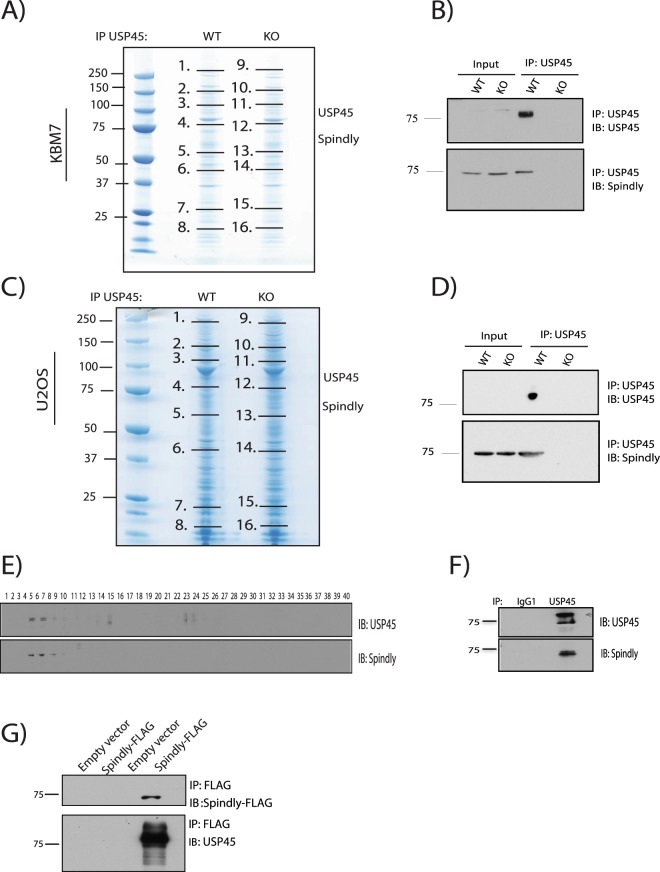
USP45 co-immunoprecipitates Spindly. (**A**) Endogenous USP45 was immunoprecipitated (IP) from wild-type (WT) or knock-out (KO) KBM7 cell lines. The immunoprecipitates were resolved on a polyacrylamide gel and stained with Coomassie. The gel was divided into the indicated sections (black lines) and proteins were identified by mass spectrometry analysis. (**B**) Endogenous USP45 was immunoprecipitated from WT or USP45 KO KBM7 cell lines and subjected to immunoblotting with indicated antibodies. (**C**) Endogenous USP45 was immunoprecipitated from WT and USP45 KO U2OS cell lines and analysed as in (**A**). (**D**) Endogenous USP45 was immunoprecipitated from WT or USP45 KO U2OS cell lines and subjected to immunoblotting with indicated antibodies. (**E**) Extracts of HEK293 cells were analysed by gel filtration on a Superdex 200 10/300 GL preparative grade column (GE Healthcare) in buffer containing 0.15 M NaCl, and every second fraction from 80 fractions obtained were denatured and analysed by immunoblotting with the indicated antibodies. (**F**) Fractions (5–10) of gel filtration analysis were used to immunoprecipitated endogenous USP45 and subjected to immunoblotting with the indicated antibodies. (**G**) Extracts of HEK293 overexpressing Spindly-FLAG were subjected to immunoprecipitation anti-FLAG and 10% of the immunoprecipitated proteins were analysed by immunoblotting for anti-FLAG and 90% for endogenous USP45. (**A**–**G**) All experiments were performed at least three times and a representative example is shown.

In order to ascertain the function of USP45, we performed immunoprecipitation assays against endogenous USP45 as has been previously described in^13^, using the same set of antibodies and cell lines (WT and Knockout (KO) of USP45). As described in^13^, the KO of USP45 was generated in KBM7 and U2OS cells by targeting exon 2 of USP45 using CRISPR/Cas9 technology as described in^27^. These cell lines were used to perform a mass spectrometry analysis to identify new potential targets of USP45. In addition to the DNA damage complex (ERCC1-XPF), where USP45 can interact with ERCC1 through its N-term domain, already described, and the proteins previously reported by Sowa *et al*., (such as RBMX, MYH10 and MYH9)^28^, we identified a new potential target of USP45 encoded by the *SPDL1* gene called Spindly or CCDC99. Spindly is a protein recruited to kinetochores in mitosis that plays a critical role in cell division, and a role for this protein in cell migration has been reported recently^26^. The protein-protein interaction result was confirmed in both KBM7 (suspension cell lines) and U2OS (adherent cell lines) cells, expressing USP45 WT but not in a USP45 KO cell lines (Fig. 1A,C and MS supp data). To validate the data of mass spectrometry, co-immunoprecipitation assays were performed using endogenous USP45 followed by immunoblot that validated the interaction between endogenous Spindly and endogenous USP45 (Fig. 1B,D).

To confirm the presence of both these proteins in the same complex we carried out a gel filtration study. Previous gel filtration studies of USP45 have established that in HEK293 cells extracts, USP45 is distributed in 2 fractions, one pool eluted in high molecular weight fractions and the other pool eluted in lower molecular weight fractions^13^. Exploring the elution profile of USP45 and Spindly we found that both proteins co-eluted in the high molecular weight fractions (fractions 5–10) (Fig. 1E). Moreover, endogenous immunoprecipitation of USP45 from these aforementioned fractions (5–10) further confirmed the presence of both proteins in a complex (Fig. 1F). Finally, a co-immunoprecipitation for Spindly from HEK293 cells overexpressing Spindly-FLAG, followed by immunoblotting for endogenous USP45, confirmed the interaction between both proteins (Fig. 1G).

### Catalytic activity of USP45 is essential to the interaction with Spindly

**Figure 2:**
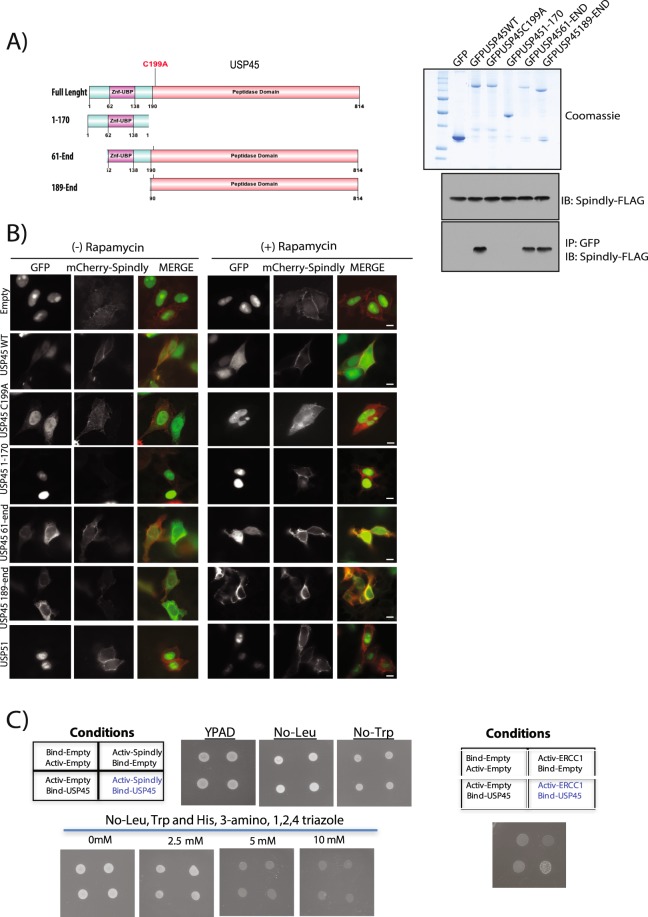
The catalytic activity of USP45 is essential for its interaction with Spindly. (**A**) U2OS cells were transiently transfected with the indicated GFP fusion constructs encoding full-length or indicated fragments of USP45 (left-hand side panel). Thirty six hours post-transfection cells were lysed and GFP immunoprecipitated. The immunoprecipitates were resolved on a polyacrylamide gel and stained with Coomassie (right-hand side upper panel) or subjected to immunoblotting with the indicated antibodies (right-hand side lower panel). GFP empty vector was used as a negative control. (**B**) U2OS cells were transfected with the indicated USP45 constructs GFP tagged, mCherrySpindly-FKBP and Lin11-FRB for 40 hours. Lin11 expression allows for mislocalisation recruitment at the plasma membrane of the FKBP-tagged protein. Rapamycin (4 μM) was administered for 1 hour (at 37 °C) and then cells were visualized under a fluorescence microscope: USP45 construct (green), Spindly (red). Left-hand side panel DMSO treated samples; right-hand side Rapamycin treated samples. 60X magnification. Scale bars: 5 μm. GFP-USP51 was used as negative control. (**C**) Yeast two-hybrid (Y2H) assays were performed with a GAL4 DNA-binding domain fusion or activation domain for each protein indicated in the table to detect interaction between the indicated proteins. Cells grown in media lacking LEU, TRP and HIS with increasing concentrations of 3-amino, 1,2,4 triazole (to select for bait and prey plasmids). (**A**–**C**) All experiments were performed at least three times and a representative example is shown.

In order to map the region of USP45 interacting with Spindly, we performed co-immunoprecipitation studies from HEK293 cells transiently expressing different USP45 fragments GFP tagged (Fig. 2A). From our data, the catalytic activity of USP45 seems to be essential; the full length USP45 protein with the catalytic cysteine mutated to alanine (C199A) was not able to interact with Spindly (Fig. 2A). In addition, a truncated version of USP45 lacking the catalytic domain (fragment 1–170) failed to interact with Spindly (Fig. 2A).

To further validate the co-immunoprecipitation result, we conducted an *“in vivo”* study exploiting the FRB-FKBP system. This method is based on the hetero-dimerization capacity of these two proteins (FRB and FKBP) only upon addition of rapamycin as was described in Putyrski *et al*.^29^ and the method section herein. Therefore, first we generated a mCherry-Spindly-FKBP tagged construct and then we co-transfected U2OS cells with three different plasmids: mCherry-Spindly-FKBP, Lin11-FRB and GFP-USP45 (separate constructs). The Lin11-FRB protein allows for conditional plasma membrane recruitment of the FKBP-tagged proteins. In the absence of rapamycin, mCherry-Spindly was distributed in the cytosol and USP45 in both cytosol and nucleus. Upon addition of rapamycin, Spindly-FKBP was recruited to the plasma membrane and colocalization with GFP-USP45 WT was observed, confirming protein-protein interaction (Fig. 2B). Interestingly, neither the catalytic inactive USP45 construct (C199A) nor the 1–170 fragment could be recruited to the plasma membrane. On the other hand, the 61-end fragment and the 189-end fragment of USP45 (both maintaining the catalytic activity intact) were positively recruited (Fig. 2B). Another DUB, USP51, was also tested as a negative control and the specificity of the USP45 interaction was confirmed (Fig. 2B). These results further underline the importance of DUB enzymatic activity in the interaction between USP45 and Spindly. Quantifications of all these assays in absence or presence of rapamycin are shown in Supp. Fig. 1.

In order to elucidate if the interaction is direct between both proteins or if they are part of the same complex but do not interact directly, we performed a yeast two hybrid assay. As described previously^13^ we used ERCC1 as a positive control for the experiment and as can be observed in Fig. 2C. Although Spindly and USP45 seems to be part of the same complex they do not interact directly.

### K48 ubiquitin chains that can be cleaved by USP45 are part of the Spindly complex

**Figure 3:**
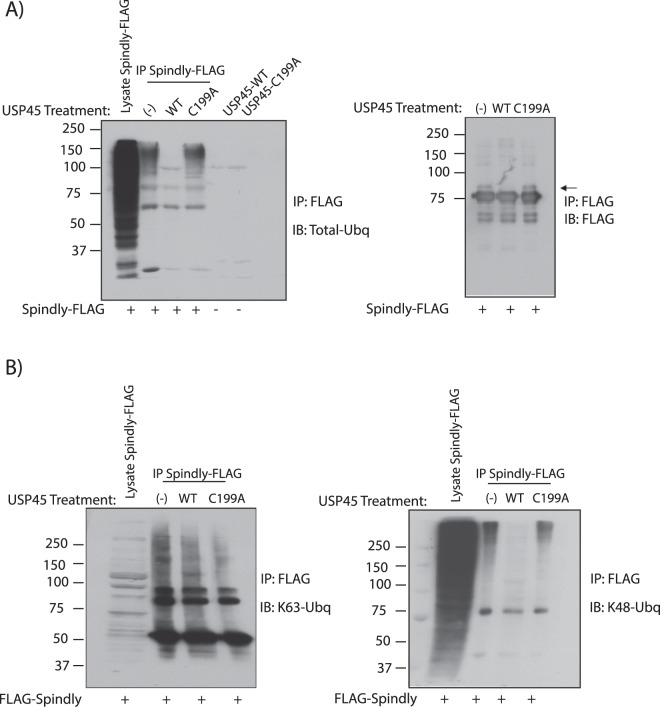
K48 ubiquitin chains that are cleaved by USP45 are part of the Spindly complex. (**A**) U2OS cells were transiently transfected with a construct encoding FLAG-Spindly. Thirty-six hours post-transfection cells were lysed and immunoprecipitation against anti-FLAG performed. The precipitates were treated in the absence or presence of 0.1 μg of recombinant USP45 wild-type (WT) or catalytic inactive USP45 (C199A). Analysis was performed by immunoblotting for total ubiquitin (Total-Ubq FK2) (left-hand side) and immunoprecipitation was confirmed by immunoblot with anti-FLAG antibody (right-hand side). (**B**) Similar experiment as in A, developed with K63 ubiquitin specific chain (left panel) and K48 ubiquitin specific chain antibody (right panel). (**A**,**B**) All experiments were performed at least three times and a representative example is shown.

Given that USP45 is a DUB, we decided to explore the ubiquitylation in the Spindly-USP45 complex. To do so, U2OS cells were transfected with Spindly-FLAG and after immunoprecipitation of the flag-tagged protein, total ubiquitin was analysed (Fig. 3A, left panel). A considerable amount of ubiquitin chains were co-immunoprecipitated with Spindly, indicative of potential Spindly ubiquitylation and/or the presence of multiple other ubiquitylated proteins in the same complex. Moreover, when these immunoprecipitated ubiquitylated proteins were treated with recombinant USP45 WT or the catalytically inactive USP45 C199A mutant, the ubiquitins were removed only by the wild-type USP45 but not by the mutant. Our data therefore indicate that USP45 could deubiquitylate Spindly and/or the other ubiquitylated proteins in the complex. Observing the Spindly immunoblot in more detail, we noticed only a small doublet of Spindly (native Spindly band plus a small higher band), but no higher molecular weight bands, so we concluded that Spindly is not polyubiquitylated under these conditions (Fig. 3A, right panel). A further validation of this data was obtained by treating cells with the proteasome inhibitor Lactacystin to test if we were able to observe Spindly polyubiquitylation; we did not see any accumulation of high molecular-weight species of Spindly in this condition (Supp. Fig. 2A). This suggests that Spindly could be potentially be mono-ubiquitylated instead of poly-ubiquitylated: Treatment of the IP of Spindly-FLAG with recombinant USP45 WT showed a removal of the potential Spindly mono-ubiquitylation band (Fig. 3A, right panel). To confirm that Spindly is a substrate of USP45 we carried out the classical ubiquitin Rhodamine (Ub-Rho110-G) assay as described in^13^ where USP45 activity can be measured. As is shown in Supp. Fig. 2B, activity of recombinant USP45 WT is impacted by addition to the reaction of the immunoprecipitated Spindly FLAG due to competition of both substrates for the enzyme activity. No activity was observed in recombinant USP45 C199A in the absence or presence of Spindly.

In order to explore ubiquitin specificity of USP45, *in vitro* de-ubiquitylation assays with 8 ubiquitin substrates were performed. Results are shown in Supp. Fig. 2C, wherein USP45 has preference for K6, K48 and K63 ubiquitin chains, limited activity on K11 and K33 and no activity against other ubiquitin types. To further analyse this specificity, we performed a time course assay with the most common studied ubiquitin types (K48 and K63) plus K6, another ubiquitin type preferred by USP45. Results shown in Supp. Fig. 2D indicate that recombinant USP45 WT is able to mediate the cleavage of 100% of the K6, K48 and K63 dimer in 60 min. As a negative control recombinant USP45 C199A was used.

To further analyse the nature of the ubiquitin chains implicated in the complex, we repeated the same experiment as in Fig. 3A, blotting in this case with a K48-specific anti-ubiquitin chain antibody or a K63-specific anti-ubiquitin chain antibody. The results indicate that ubiquitin chains of K63 ubiquitin can immunoprecipitate with Spindly but they are not affected by USP45 activity whereas K48 specific ubiquitin chains not only immunoprecipitate with Spindly but also can be processed by enzymatically active USP45 but not by the inactive form (Fig. 3B). We cannot exclude the presence of K6 ubiquitin chains implicated in the complex as well, due to the USP45 specificity demonstrated against them, but the lack of commercial antibodies against K6 impairs further exploration of this^30^. Since we already confirmed that Spindly was not poly-ubiquitylated in K48 chains (Supp. Fig. 2A), we performed several follow up experiments to search for potential Spindly interactors as well as kinetochore proteins (the site where Spindly has been described to have its main function), these proteins included Dynein (Dynein intermediate chain, DIC), Dynactin (p50), RZZ complex (ZW10 and KNTC1). In all cases, all proteins were analysed after Spindly IP in absence or presence of recombinant USP45 WT or catalytic inactive protein. However, to date, we have been unable to identify the protein of the complex that is polyubiquitylated in K48 ubiquitin type chains (Supp. Fig. 3A).

### USP45 is a DUB that catalyses the de-monoubiquitylation of Spindly

**Figure 4:**
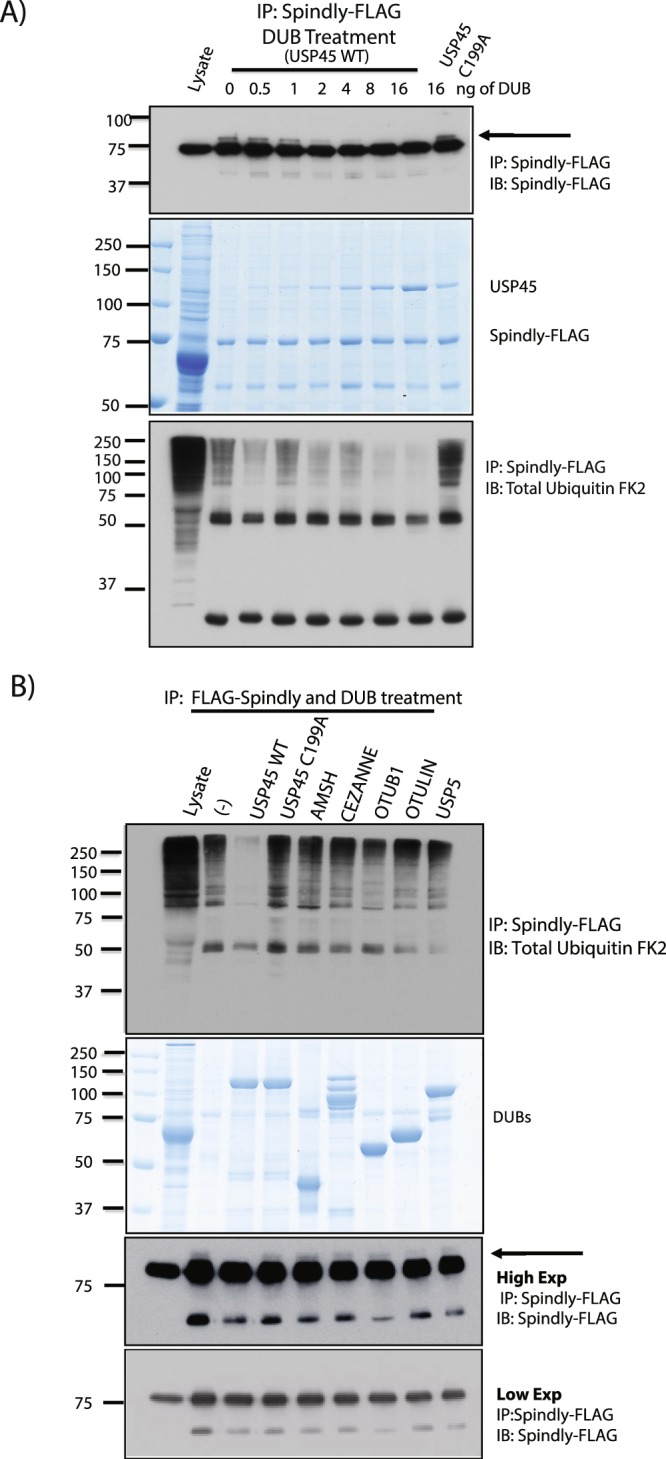
USP45 is a DUB that catalyses the di-monoubiquitylation of Spindly. (**A**) HEK293 cells were transfected with Spindly-FLAG. Spindly-FLAG was immunoprecipitated and treated in the absence or presence of increasing concentration of recombinant USP45 wild-type (WT) or catalytic inactive USP45 (C199A) at highest concentration. The immunoprecipitates were resolved on a polyacrylamide gel and subjected to immunoblotting with the indicated antibodies (upper and lower panels) or stained with Coomassie (middle panel). (**B**) HEK293 cells were transfected with Spindly-FLAG. Spindly-FLAG was immunoprecipitated and treated with several different DUBs. The immunoprecipitates were resolved on a polyacrylamide gel and subjected to immunoblotting with the indicated antibodies (upper and lower panel) or stained with Coomassie (middle panel). (**A**,**B**) All experiments were performed at least three times and a representative example is shown.

Monoubiquitylation is a protein modification that occurs when a single ubiquitin is bound to a single lysine on the target protein. This process has been reported to be independent from degradation. To explore further if the monoubiquitylation of Spindly observed in Fig. 3A is real and if it is modulated by USP45, we performed an immunoprecipitation assay of FLAG-Spindly and we treated the beads with increasing amounts of recombinant USP45 WT, from 0 to 16 ng. We observed that 2 ng of recombinant USP45 WT was sufficient to remove the monoubiquitylation of Spindly. As a negative control, we used the catalytically inactive USP45 C199A mutant at maximum concentration (16 ng) and we did not observe any removal of Spindly monoubiquitylation (Fig. 4A). To cleave total ubiquitin associated with the Spindly complex, higher amounts of recombinant USP45 WT were necessary; again as a negative control USP45 C199A mutant was used.

To test the specificity of USP45 in the process we did a similar assay using a panel of DUBs from different families. After immunoprecipitation of Spindly, beads were treated with recombinant USP45 WT and C199A, and with DUBS with different specific preference of ubiquitin chain type cleavage, such as AMSH (K63), CEZANNE (K11), OTUB1 (K48) and OTULIN (Linear chains). Immunoblots for total ubiquitin showed that ubiquitin chains associated with Spindly can be cleaved only by active USP45 as well as the mono-ubiquitin observed on Spindly after immunoprecipitation (Fig. 4B).

In order to identify the lysine mono-ubiquitylated in Spindly we performed a mass spec analysis with a Gly-Gly antibody and 2 lysines were identified as a potential target sites in peptides analysed (MS supp data). We generated single and double mutants of these potential sites: residues 115 and 117 to arginine, but we failed in the specific identification of the site, probably because other lysines can be ubiquitylated in absence of these specific ones (Supp. Fig. 4). It is important to mention that Spindly protein contains 62 lysine residues in its sequence.

### USP45 has a role in cell migration

**Figure 5:**
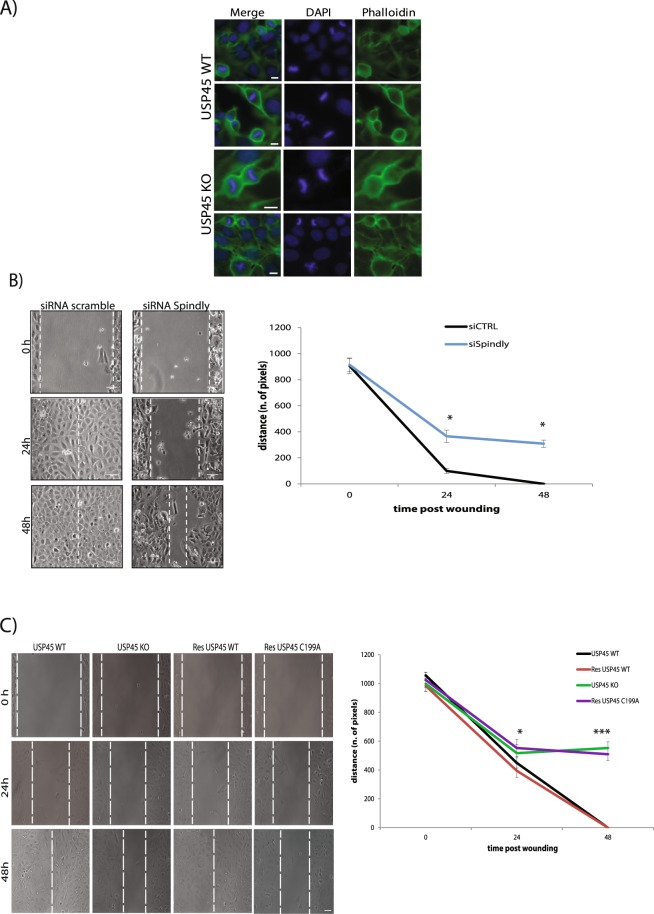
Silencing of USP45 or Spindly leads to defects in cell migration. (**A**) U2OS cell lines either expressing USP45 WT or KO were analysed for cytokinesis rate. The figure shows that the lack of USP45 does not affect the cytokinesis process and cells division as show the DAPI staining. Staining as follow: Actin (Phalloidin, green), nuclei (DAPI, blue). 60X magnification. Scale bars: 5 μm. (**B**) Images from time-lapse analysis of wound healing assay of U2OS cells treated either with siRNA control (scramble) or siRNA for Spindly (siRNA Spindly). Dotted white lines indicate the edge of the wound. Three independent experiments were performed and data in the graph represent mean ± s.d. Scale bars: 10 μm. (**A**,**B**) All experiments were performed at least three times and a representative example is shown. (**C**) USP45 WT or KO U2OS cells. Transient transfection of USP45 KO U2OS cells with USP45 WT construct rescued the delay defects; while instead transient transfection of KO U2OS cells with catalytic inactive USP45 (C199A) construct maintain the defect. Dotted white lines indicate the edge of the wound. Three independent experiments were performed and data in the graph represent mean ± s.d. Student t-test was used to determine the statistical significance. Scale bars: 10 μm.

To understand in what biological process these two proteins could be interacting, first we analysed the levels of phospho-H3, a marker for mitotic cells in WT and USP45 KO U2OS cell lines. Spindly has been described as an active player in cell division, acting at kinetochores in complex with other proteins^15,19^. Our results however indicate that the absence of USP45 does not affect mitotic progression and therefore the role of USP45 role is probably not related to Spindly in mitosis (Supp. Fig. 5). In addition, both USP45 WT and USP45 KO cells have a similar cell cycle profile as described in^13^. We also analysed the rate of cytokinesis in cells depleted of USP45. The process of cytokinesis can be followed by staining actin with phalloidin and following the formation of the actin ring over time. However, upon USP45 KO cytokinesis was also not altered, confirming that neither mitosis nor proliferation rate is affected by the lack of USP45 at 48 hours; results are shown in Fig. 5A.

Recent studies have shown that Spindly is required for proper cell migration, with its depletion causing defects in wound closure in a cell monolayer. Moreover, Spindly localises on microtubule tips and at focal adhesions suggesting a role in cell migration associated with the dynein-dynactin complex, with which it was already shown to interact^26,31,32^. Thus, we explored the potential role of USP45 in this process by performing the same wound-closure experiments as for Spindly depleted cells, using USP45 KO cells. We generated a wound in a monolayer of cells to observe the edge driven movement promoted to close the gap (“wound”). The wound was generated by the use of a silicon chamber divided in the middle by a wall. Cells were seeded in the two wells and once confluence was reached the insert was removed, generating a reproducible gap in the monolayer of cells. Our data in USP45 KO cells showed similar results to what was obtained in cells lacking Spindly (Fig. 5B). USP45 depleted cells were defective in cell migration and the defects observed were not produced by off-target effects: phenotypic rescue could be achieved only by re-expressing the catalytically active USP45 and not with the inactive mutant C199A, suggesting a potential interplay of Spindly and USP45 in cell migration (Fig. 5C).

## Discussion

Spindly is a key regulator of mitosis, due to its role in recruiting the dynein dynactin complex to kinetochores and its ability to activate the motility of the supercomplex. Recent work showed that C-terminal farnesylation of the protein is essential for these functions^21–23^. In this manuscript, we show that Spindly can be mono-ubiquitylated and associates with both a DUB protein, USP45, and a large complex of ubiquitylated proteins.

**Figure 6:**
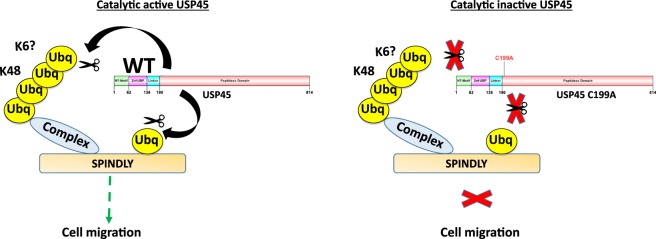
Model of USP45 and Spindly interaction. Proposed model illustrating the interaction between USP45 and Spindly. Interaction is dependent on USP45 catalytic activity. Spindly is mono-ubiquitylated and this ubiquitin can be removed by active USP45. K48 ubiquitylated complex that interacts with Spindly is also de-ubiquitylated by USP45. In the absence of USP45 catalytic activity, interaction is abolished and cell migration is affected similarly to the phenotype described for lack of Spindly.

USP45 is a DUB with preference for K6, K48 and K63 ubiquitin chains, at least *in vitro* and limited activity is observed on K11 and K33 and no activity against other ubiquitin types. Moreover, here we described that USP45 can both deubiquitylate Spindly and remove the K48 chains from proteins in the Spindly-binding complex and potentially K6 chains although currently available tools impairs the ability to explore this. Interestingly, the catalytic activity of USP45 is required for the interaction between the DUB and the Spindly complex, which is highly unusual for DUB-target protein interactions as most DUBs recognize their substrates through non-catalytic regions. This could be explained if the interaction is through the ubiquitin motif as data from the yeast two hybrid assay reveals that the interaction between USP45 and Spindly is not direct or could be through a third, unknown ubiquitylated protein in the complex. This raises the question of whether USP45 needs to remove an ubiquitin or ubiquitin chain in order to recognize its binding partner. Given that the catalytically dead mutant cannot bind to the Spindly complex we favour this hypothesis for how USP45 interacts with the high-molecular weight ubiquitylated complex. Our proposed model of the hypothesis is summarized in Fig. 6.

In spite of our efforts to identify the ubiquitylated site of Spindly and potential candidate lysines identified by mass spectrometry and mutation of residues 115 and 117 to arginine, so far we have been unable to find the specific site of modification. These residues, both are in the first Coil Coil Domain I of Spindly, which has been described as a region dispensable for its kinetochore localization^23^, these data correlate with our data that demonstrate the lack of effect of USP45 in kinetochore formation or cell division, as discussed below. We hypothesise that in the absence of the normal target lysine in our mutant models, additional lysines within Spindly may undergo ubiquitylation and it is noted that the high number of lysine residues (62 within the Spindly sequence), provide some rationale for this proposal.

Curiously, USP45 activity does not appear to be required for the activity of Spindly at the kinetochore. USP45 KO cells divide without any apparent delay. However, we cannot rule out that there is another DUB that can compensate for the lack of USP45 in these cells. We did find a phenotypic overlap between Spindly and USP45 in cell migration. The loss of either protein slows wound closure in a 2D cell migration model. Further research will be needed to definitively identify the mechanism by which USP45 affects migration and determine whether it is directly regulating Spindly in this behaviour.

## Materials and Methods

### Reagents

Ubiquitin monomer, BSA, Tris and DTT were purchased from Sigma Aldrich. Di-Ubiquitin topoisomers (linear, K6, K11, K27, K29, K33, K48 and K63-linked ubiquitin-ubiquitin) were purchased from Boston Biochem. Complete protease inhibitors cocktail tablets were obtained from Roche. Protein G–Sepharose, glutathione–Sepharose and ECL reagents were from GE Healthcare. Doxycycline, DMSO, BSA, 3-amino, 1,2,4 triazole and benzamidine were from Sigma–Aldrich. PMSF was from Melford. Novex 4–12% polyacrylamide Bis-Tris gels, LDS sample buffer, puromycin, hygromycin, blasticidin and other tissue culture reagents were from Invitrogen Life Technologies. Instant Blue Coomassie stain was from Expedeon. PEI (polyethylenimine) was from Polysciences.

### General methods

Standard protocols were used for tissue culture, electrophoresis, immunoblotting and immunoprecipitation as well as DNA procedures. The QuikChange® site-directed mutagenesis method (Stratagene) with KOD polymerase (Novagen) was used to generate mutants. DNA plasmids were purified using QIAGEN maxi-prep kits according to the manufacturer’s protocol from Escherichia coli DH5α cells. All DNA plasmids were sequenced by the Sequencing Service (MRC Protein Phosphorylation Unit, College of Life Sciences, University of Dundee, Dundee, U.K.; http://www.dnaseq.co.uk), using DYEnamic ET terminator chemistry (GE Healthcare) on Applied Biosystems automated DNA sequencers.

### Antibodies

The following antibodies were raised by the Division of Signal Transduction Therapy (DSTT) at the University of Dundee in sheep and affinity-purified against the indicated antigens: anti-USP45 (1st, 2nd and 3rd bleed of S052D, residues 756–780 of human USP45) and (1st, 2nd and 3rd bleed of S109D, residues 29–80 of human USP45). Antibody against FLAG M2 clone was from Sigma Aldrich. Total FK2 ubiquitin antibody and K48-linkage-specific ubiquitin antibodies (clone Apu2) was from Millipore and K63-linkage-specific ubiquitin antibodies (D7A11) were from Cell Signaling. Secondary antibodies coupled to horseradish peroxidase were from Thermo Scientific.

### Cell culture, treatments and cell lysis

DMEM (Dulbecco’s modified Eagle’s medium) supplemented with 10% FBS, 2 mM glutamine and 1x antibacterial/antimycotic solution was used to culture U2OS (human osteosarcoma cells) and HEK-293 (human embryonic kidney cells). Transient transfections of HEK-293 or U2OS cells were carried out using PEI from Polysciences. Lysis buffer containing 50 mM Tris/HCl (pH 7.5), 1 mM EGTA, 1 mM EDTA, 1% Triton X-100, 50 mM NaF, 10 mM sodium 2-glycerophosphate, 5 mM sodium pyrophosphate, 1 mM sodium orthovanadate, 0.27 M sucrose, 1 mM benzamidine (added before lysis), 1 mM PMSF (added before lysis) and 0.1% 2-mercaptoethanol (added before lysis) was used to lyse cells. To observe ubiquitylation in immunoblotting, cells were lysed in lysis buffer containing 20 mM NEM (Sigma Aldrich). Lysates were clarified by centrifugation at 16000 g for 15 min at 4 °C and either used for further experiments or frozen in liquid nitrogen and stored at −80 °C. Protein determination was performed using Bradford method with BSA as a standard. Lactacystin (Sigma Aldrich) was used at 5 µM for 6 hours.

### Expression of DUB enzymes

For insect cell protein expression, appropriate cDNAs were cloned into the pFastBac vector, baculoviruses were generated to encode various Dac-tagged DUB enzymes. Sf21 cells were typically infected with P1 virus stocks 120 and harvested 48 hours later. Cells were lysed in Dac lysis buffer (40 mM Tris pH 7.5, 0.2% Triton-X 100, 0.5 mM EGTA, 0.1 mM EDTA, 1 mM DTT) supplemented with 1 mM Pefabloc and Leupeptin at 20 μg/ml then centrifuged to remove insoluble material. For protein purification, supernatants were subjected to affinity chromatography using ampicillin-Sepharose resin. The DUB enzymes were either eluted from the ampicillin-Sepharose by incubating 4x for 15 min with 1 resin volume of 50 mM Tris pH 7.5, 5% v/v glycerol, 100 mM NaCl, 10 mM ampicillin, 1 mM DTT, 0.03% Brij-35 or recovered by digesting the DUB off the Dac-tag using TEV-protease in 50 mM Tris pH 7.5, 100 mM NaCl, 0.03% Brij 35.

### *In vitro* DUB assay

DUB *in vitro* activity was performed using the indicated concentrations of each DUB in bis-tris acrylamide gels. Both enzymes and immunoprecipitations substrates were freshly mixed in reaction buffer (40 mM Tris-HCl, pH 7.6, 5 mM DTT, 0.005% BSA) for each run. The reaction mixture was incubated at 30 °C for 60 minutes. The reaction was stopped by adding the SDS loading buffer if the sample was used to run a bis-acrylamide gel.

### Immunoblotting

Lysates (20 μg) were heated in SDS sample buffer and subjected to SDS-PAGE following by an electrotransfer to nitrocellulose membranes. Membranes were blocked in 50 mM Tris-HCl pH 7.5, 0.15 mM NaCl and 0.1% Tween (TBST) containing 5% skimmed milk for 1 hour. The membranes were then probed with the primary antibody (1 μg/ml for the sheep antibodies or 1000-fold dilution for the commercial antibodies) for 16 hours at 4 °C in TBST containing 5% skimmed milk (sheep antibodies) or 5% BSA (commercial antibodies). Detection of protein was performed using horseradish peroxidase-conjugated secondary antibodies and enhanced chemiluminescence reagent.

### Immunoprecipitation

Lysates (1–2 mg) were incubated at 4 °C for 1 hour on a shaking platform with 5 μl of protein G-Sepharose coupled to USP45 or commercial FLAG-beads. The immunoprecipitates were washed twice with 1 ml of lysis buffer containing 0.5 M NaCl and twice with buffer A (50 mM Tris-HCl pH 7.5, 0.1 mM EGTA and 0.1% 2-mercaptoethanol).

### Identification of USP45 interacting protein by mass spectrometry

50 mg of cell lysates were pre-cleared by incubation with 100 μl of pre-immune IgG covalently coupled to protein G-Sepharose for 1 hour at 4 °C on a rolling shaker. The supernatants were then incubated with 50 μg of anti-USP45 specific antibody covalently coupled to protein G-Sepharose for 1 hour at 4 °C on a rolling shaker. The immunoprecipitates were washed three times with 10 ml of lysis buffer containing 0.5 M NaCl and twice with 10 ml of buffer A. The beads were re-suspended in a total volume of 30 μl of LDS sample buffer (Invitrogen). The samples were then filtered with a 0.44 μm Spin-X filter (Corning), reduced with 10 mM dithiothreitol, boiled and subjected to electrophoresis on a NuPAGE Bis-Tris 4–12% polyacrylamide gel. Colloidal Coomassie stained gel was divided as into piece, each of which were washed with 0.1 M NH_4_HCO_3_ and 50% acetonitrile/50 mM NH_4_HCO_3_, alkylated with 50 mM iodoacetamide in 0.1 M NH_4_HCO_3_ (30 min at room temperature), washed as above, dried, and incubated with 25 mM triethylammonium bicarbonate with 5 μg/ml trypsin overnight at 30 °C on shaker. The resultants peptides were submitted to LC-MS on a Proxeon EASY-nLC nano liquid chromatography system coupled to a Thermo-LTQ-Orbitrap mass spectrometer. Data files were searched against the SwissProt mouse database using Mascot (http://www.matrixsciences.com) run on an in-house system, with 10 p.p.m. mass accuracy for precursor ions and a 0.8 Da tolerance for fragment ions.

### Rapamacyin mislocalisation assay

Plasmids used for this assay were generated as follows: the FKBP coding sequence was inserted before the stop codon of the Spindly gene using the In-Fusion cloning method (Clontech). The mcherry-Spindly plasmid was linearised with the primers TAAGCGGCCGCTCGAGTCTAGAGG and CTGTTGAGGGCACTGGGTCTCTGG, and FKBP was amplified with the primers CAGTGCCCTCAACAGGGAGTGCAGGTGGAGACTATCTCC and TCGAGCGGCCGCTTATTCCAGTTTTAGAAGCTCCACATCG in which the underlined regions are homologous to the breakpoints in the linearised mcherry-Spindly plasmid. After the ligation reaction, colonies were screened by PCR and confirmed by sequencing. The Lin11-FRB plasmid was a kind gift of Dr. A. Saurin.

U2OS cells were transfected with the FRB and FKBP plasmids and simultaneously with the GFP- target protein. After 24 hours, rapamycin was added at 4 µM and incubated for between 30 minutes and 4 hours. Cells were then fixed with a 4% PFA solution, obtained by diluting Paraformaldehyde 32% Solution (EM Grade) in PHEM 1X (60 mM PIPES, pH 6.9; 25 mM HEPES; 10 mM EDTA; 2 mM MgCl 2x 6H_2_O), for 10 minutes at room temperature. Slides were washed in PHEM-wash (PHEM + 0.1% Triton X-100), permeabilized in PHEM-T (PHEM + 0.5% Triton X-100) for 5 minutes and fixed again in 4% PFA for 10 minutes. After three washes in PHEM-wash slides were blocked in Abdil (TBS-0.1% Tween, 0.1% Azide, 2% BSA) for 1 hour before incubating for one hour with DAPI (1:500) to stain DNA. Slides were finally mounted using DAKO Mounting Media (Agilent Technologies).

High-resolution images were collected with an imaging system (DeltaVision Elite; GE Lifesciences) using either a 60x or a 100x objective lens.

Images were analysed using FiJi Software. Measurements were conducted by drawing a line across the two edges of a cell and measuring the fluorescence intensity for both channels (green (GFP) and red (mCherry)). Graphs represent the average of data from three independent experiments for which at least 50 cells were measured each time.

### Immunofluorescence

PFA 4% solution was obtained by diluting Paraformaldehyde 32% Solution (VWR) in PHEM 1X (60 mM PIPES, pH 6.9; 25 mM HEPES; 10 mM EDTA; 2 mM MgCl 2x 6H_2_O). Cells were seeded onto sterilised glass coverslips (Menzel-Glaser). When confluent, cells were fixed in 4% PFA for 10 minutes at room temperature, washed in PHEM-wash (PHEM + 0.1% Triton X-100), permeabilized in PHEM-T (PHEM + 0.5% Triton X-100) for 5 minutes and fixed again in 4% PFA for 10 minutes. After three washes in PHEM-wash they were blocked in AbDil buffer (TBS-0.1% Tween, 0.1% Azide, 2% BSA) for 1 hour. At this point slides were incubated with primary antibodies for one hour at room temperature or overnight at 4 °C.

After four washes in PHEM-wash, the coverslips were incubated for one hour with secondary (either Alexa or Jackson) antibodies diluted in AbDil buffer (1:500) and the DNA was stained with DAPI (Sigma) (1:500). Coverslips were washed three times in PHEM-wash and once in PHEM 1X prior to being mounted onto VWR SuperPremium Microscope Slides in DAKO mounting media (Agilent Technologies). Slides were finished with nail polish and allowed to dry before imaging was performed. High-resolution images were collected with an imaging system (DeltaVision Restoration; Applied Precision) using a 60X/1.35 oil (Olympus) objective lens. Images were then processed using FiJi software.

### Cell migration assay and scratch assay

U2OS cells were seeded and cultured in a Silicone Culture-Insert (ibidi; Matinsried, Germany) set into a 35 mm µ-Dish (ibidi) and grown for at least 24 hours. Once cells reached confluence, the insert was removed and cells were washed once with fresh media, leaving the cells to migrate for the indicated times. For the rescue experiments, U2OS cells were transiently transfected with USP45 WT or KO. Imaging was performed on a Nikon Ti microscope (Nikon, Tokyo, Japan) fitted with an OKOlab environmental control chamber (Okolab, Pozzuoli, Italy), 20x 0.45NA objective, Nikon 421 PerfectFocus System, and a Photometrics Cascade II camera (Tucson, AZ, USA) and NIS Elements software. Images were then processed using FiJi software. Measurements were made by drawing a line between the edges and measuring the distance at the indicated time points. Kymograph analyses were conducted using Volocity software (Perkin Elmer; 425 Waltham, MA, USA) to measure the velocity of movement over the time.

### Yeast two-hybrid assay

Yeast strain used was AH109 (Invitrogen) which has a HIS3 gene regulated by Gal4-binding sites. This strain was transformed with a pAS2.6 plasmid (Invitrogen) encoding USP45 fused to the DNA-binding domain of Gal4 (the plasmid also has the TRP1 selectable marker) and with pACT2.6 plasmids (Invitrogen) encoding Spindly or ERCC1 fused to the Gal4 activation domain. As a negative control empty vectors were used. Cells were plated in synthetic complete media lacking LEU and TRP and histidine (HIS), as well as in minimal medium lacking leucine (LEU) and tryptophan (TRP) to assay reporter gene activation. Colony growth was observed in each condition.

### Rhodamine DUB assay

This assay was performed using rhodamine 110-glycine and 20 ng/μl of USP45 WT or catalytic inactive C199A at 30 °C for 1 hour in 40 mM Tris–HCl buffer at pH 7.6, with 5 mM DTT and 0.05 mg/ml BSA. Samples were prepared in triplicates and analysed in 96-well plates using a Perkin Elmer Envision 2104 multi-label reader at Excitation/Emission 485/535 nm.

### *In vitro* DUB assays

DUB *in vitro* activity was performed using 50 ng of each DUB. Enzymes and substrates were freshly mixed in reaction buffer (40 mM Tris–HCl, pH 7.6, 5 mM DTT, 0.005% (w/v) BSA for each run. The reaction mixture was incubated at 30 °C for 60 min. The reaction was stopped by adding the SDS loading buffer when the sample was used to run a 15% (w/v) bis-acrylamide gel. For figure legends I would add:

### Statistics

Student’s t-test was used to determine the statistical significance of rhodamine and H3 assay as well as migration speeds and wound closure dimensions.

## Electronic supplementary material

Supp Info legeds and supp Figures

Original blots

Mass spec Support data
