# Supplementary material for: USP45 and Spindly are part of the same complex implicated in cell migration
Source: Sci Rep. 2018 Sep 26;8:14375. doi: 10.1038/s41598-018-32685-8 (PMC6158257; doi:10.1038/s41598-018-32685-8)

USP45 and Spindly are part of the same complex implicated in cell migration

Claudia Conte[1,&], Eric R. Griffis[1, ¶], Ian Hickson[2,$], Ana B. Perez-Oliva[1*]

Supplementary Figures

Supplemental Figure 1. Quantification of FRB-FKBP system.
Measurements conducted for USP45 mis-localisation recruitment at the plasma membrane; values were obtained by drawing a line between the two edges of the cell and then the fluorescence intensity was measured using a plug-in in FiJi (multichannel plug-in) and plotted with Excel. Similar measurements were conducted for all the USP45 mutants tested. For each experiment 50 cells were measured and then the average values for each channel were plotted. All experiments were performed at least three times and a representative example is shown.

Supplemental Figure 2. Analysis of polyubiquitylation of Spindly and USP45 ubiquitin type specificity.
(A) HEK293 cells treated with lactacystin (5µM) for 6 hours and lysed with a buffer containing 0.1 M N-ethylmaleimide. Samples lysated to explore polyubiquitylation of Spindly. Immunoblotting with total ubiquitin antibody and Spindly.
(B) Ability of bacterial expressed recombinant full length USP45 WT and USP45 mutant C199A to hydrolyse Ub-Rho110-G was assessed in presence and absence of immunoprecipitated Spindly-FLAG. Values determined at t = 60 min were plotted. Three independent experiments were performed and data in the graph represent means ± s.d. Student t-test was used to determine the statistical significance.
(C) Ability of recombinant USP45 WT and C199A mutant to cleave 8 dimers of ubiquitin at t = 60min.
(D) Time course activity of USP45 WT or catalytic inactive C199A at time indicated against K6, K48 and K63 ubiquitin dimers.
(A-D) All experiments were performed at least three times and a representative example is shown.

Supplemental Figure 3. Analysis of polyubiquitylation of Spindly interactors.
HEK293 cells were transfected with Spindly-FLAG. Spindly-FLAG was immunoprecipitated and treated in the absence or presence of 10 ng of recombinant USP45 wild-type (WT) or catalytic inactive USP45 (C199A). The immunoprecipitates were resolved on a polyacrylamide gel and subjected to immunoblotting with the indicated antibodies. Arrows indicate the molecular weight of each protein. Non-specific bands are indicated with *. All experiments were performed at least three times and a representative example is shown.

Supplemental Figure 4. Mutants to explore site of mono-ubiquitylation of Spindly.

HEK293 cells transfected with FLAG constructs: Spindly WT or single mutant K115R or K117R or double mutant (K115R and K117R) were analysed by immunoblotting with FLAG monoclonal antibody. All experiments were performed at least three times and a representative example is shown.

Supplemental Figure 5. USP45 does not play a role in mitotic division.

U2OS cell lines expressing USP45 WT or KO were analysed for positive expression of phospho-H3 as a marker for mitotic division. The figure indicates that the absence of USP45 does not affect the presence of phospho-H3 as indicated from the % of total of cells positive for phospho-H3 (no significance difference). Three independent experiments were performed and data in the graph represent means ± s.d. Student t-test was used to determine the statistical significance. Scale bars: 5μm.

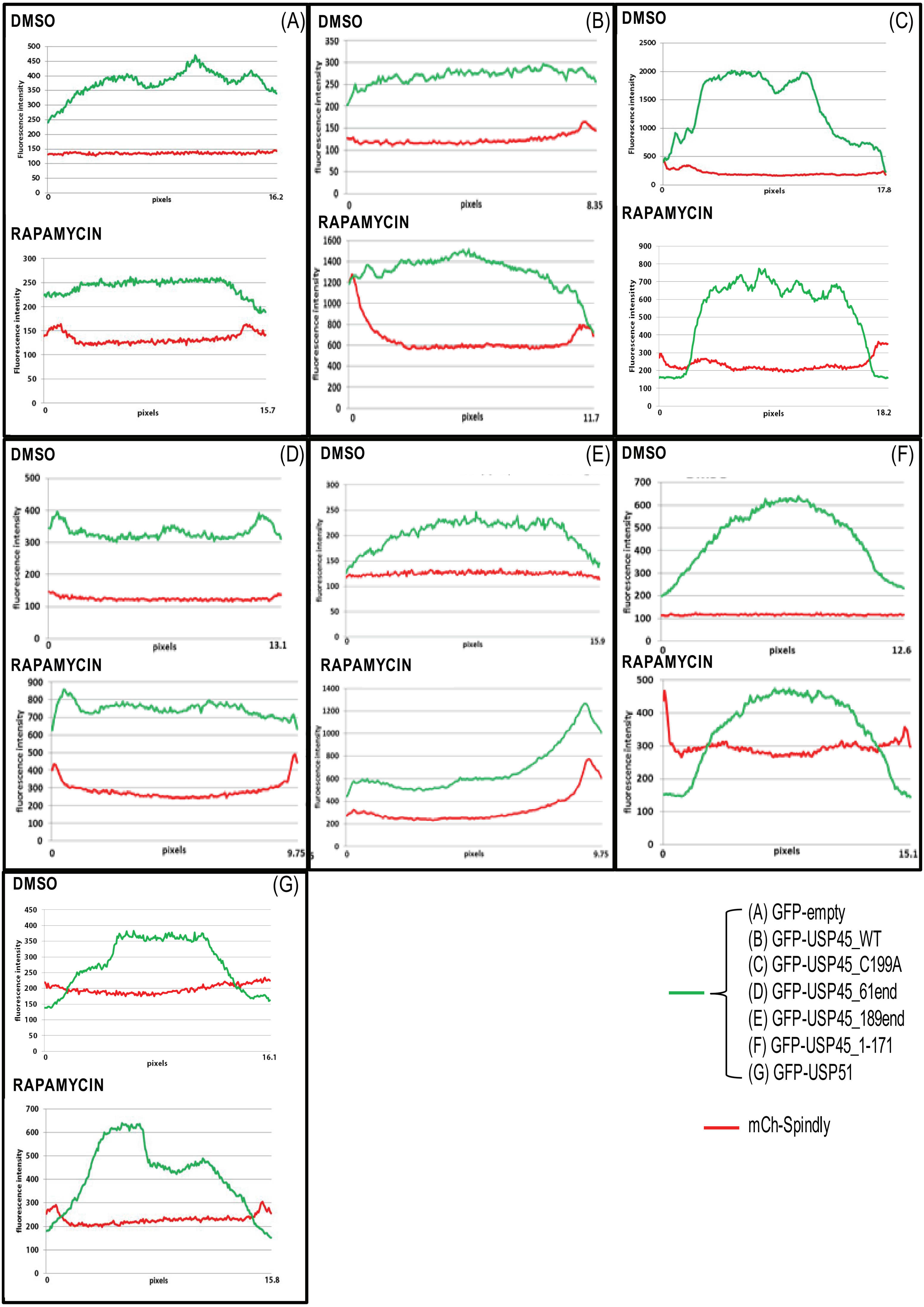
(A)
DMSO
RAPAMYCIN
Fluorescence intensity
pixels
16.2
15.7
(B)
DMSO
RAPAMYCIN
fluorescence intensity
pixels
8.35
11.7
(C)
DMSO
RAPAMYCIN
Fluorescence intensity
pixels
17.8
18.2
(D)
DMSO
RAPAMYCIN
fluorescence intensity
pixels
13.1
9.75
(E)
DMSO
RAPAMYCIN
fluorescence intensity
pixels
15.9
9.75
(F)
DMSO
RAPAMYCIN
fluorescence intensity
pixels
12.6
15.1
(G)
DMSO
RAPAMYCIN
fluorescence intensity
pixels
16.1
15.8
(A) GFP-empty
(B) GFP-USP45_WT
(C) GFP-USP45_C199A
(D) GFP-USP45_61end
(E) GFP-USP45_189end
(F) GFP-USP45_1-171
(G) GFP-USP51
mCh-Spindly

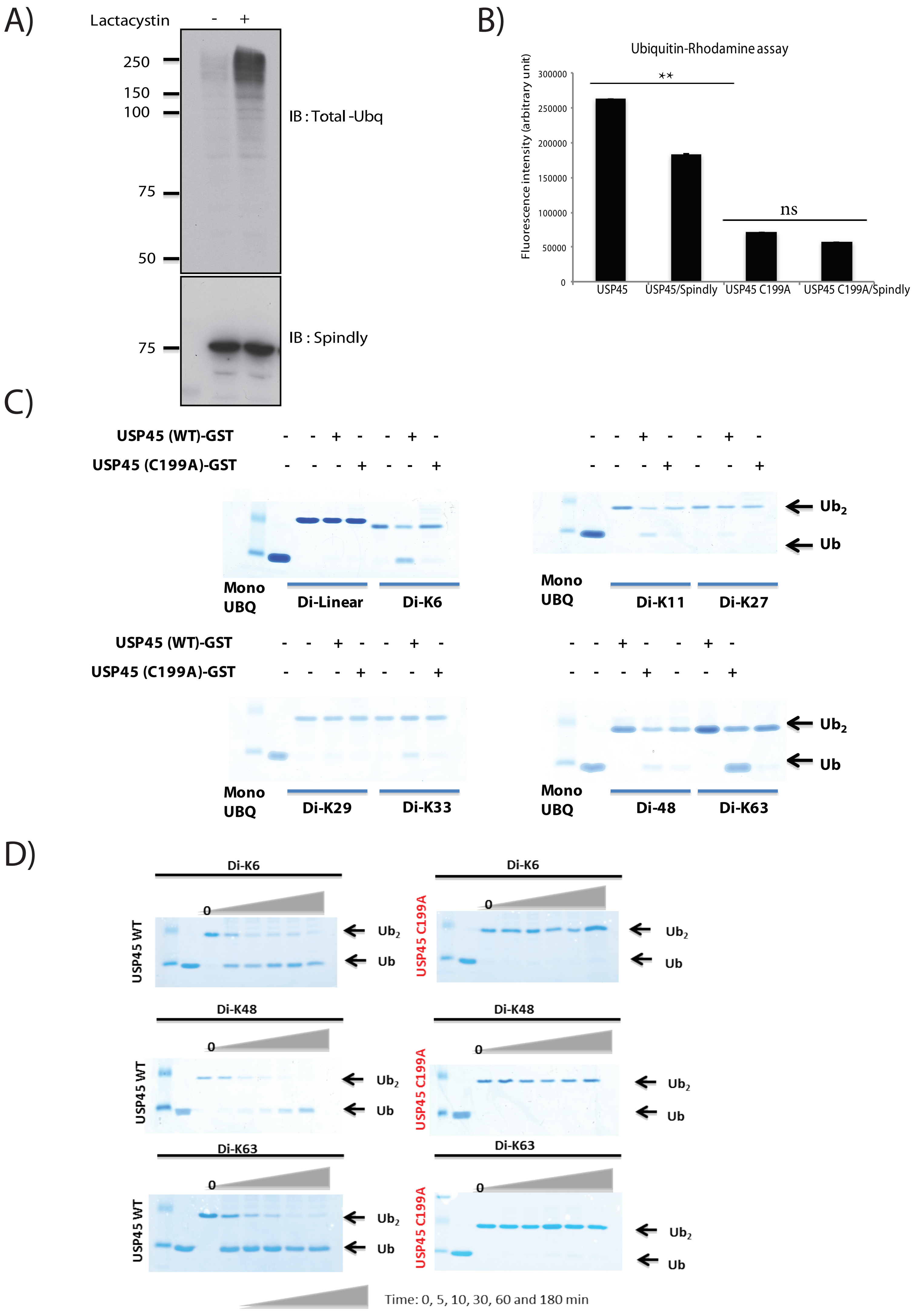
A)
Lactacystin - +
250
150
100
75
50
IB : Total -Ubq
75
IB : Spindly
B)
Ubiquitin-Rhodamine assay
Fluorescence intensity (arbitrary unit)
300000
250000
200000
150000
100000
50000
0
**
ns
USP45
USP45/Spindly
USP45 C199A
USP45 C199A/Spindly
C)
USP45 (WT)-GST
USP45 (C199A)-GST
Ub2
Ub
Mono UBQ
Di-Linear
Di-K6
Di-K11
Di-K27
Di-K29
Di-K33
Di-48
Di-K63
D)
Di-K6
Di-K48
Di-K63
USP45 WT
USP45 C199A
0
Ub2
Ub
Time: 0, 5, 10, 30, 60 and 180 min

A)

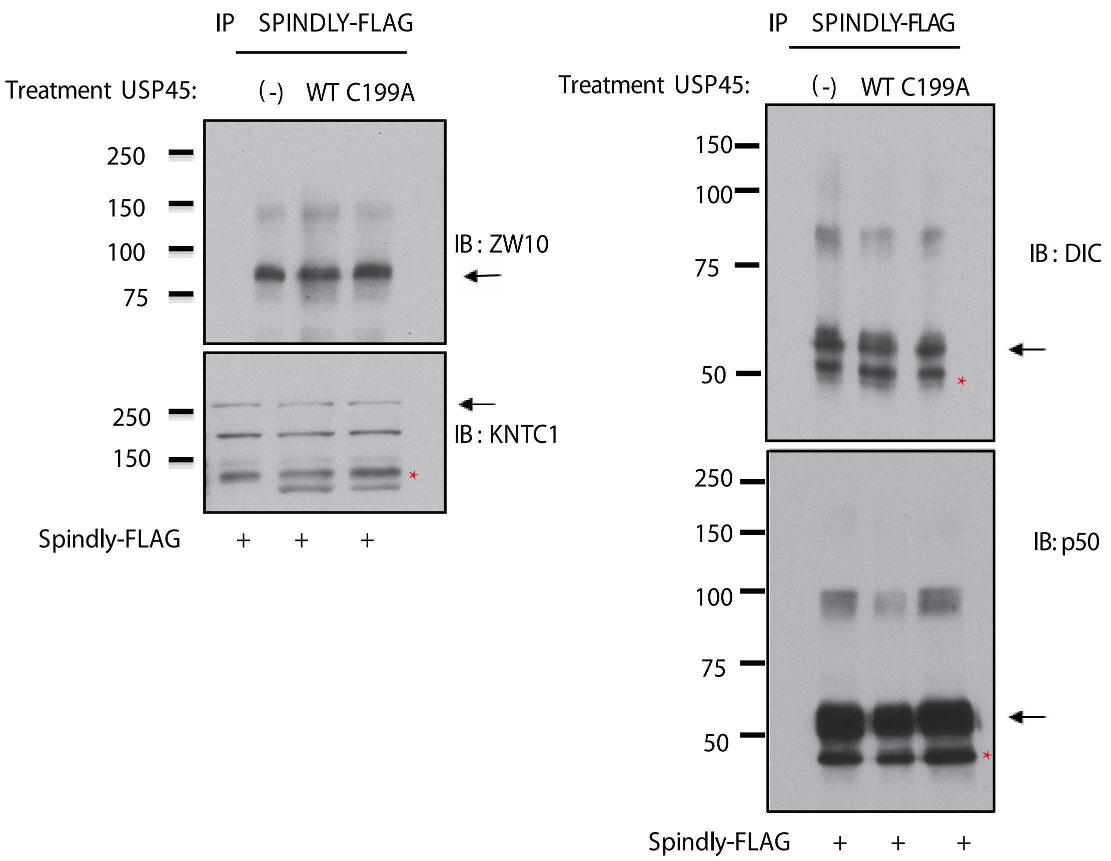
IP SPINDLY-FLAG
Treatment USP45: (-) WT C199A
250
150
100
75
IB : ZW10
250
150
IB : KNTC1
Spindly-FLAG + + +
IP SPINDLY-FLAG
Treatment USP45: (-) WT C199A
150
100
75
50
IB : DIC
250
150
100
75
50
IB: p50
Spindly-FLAG + + +

A)

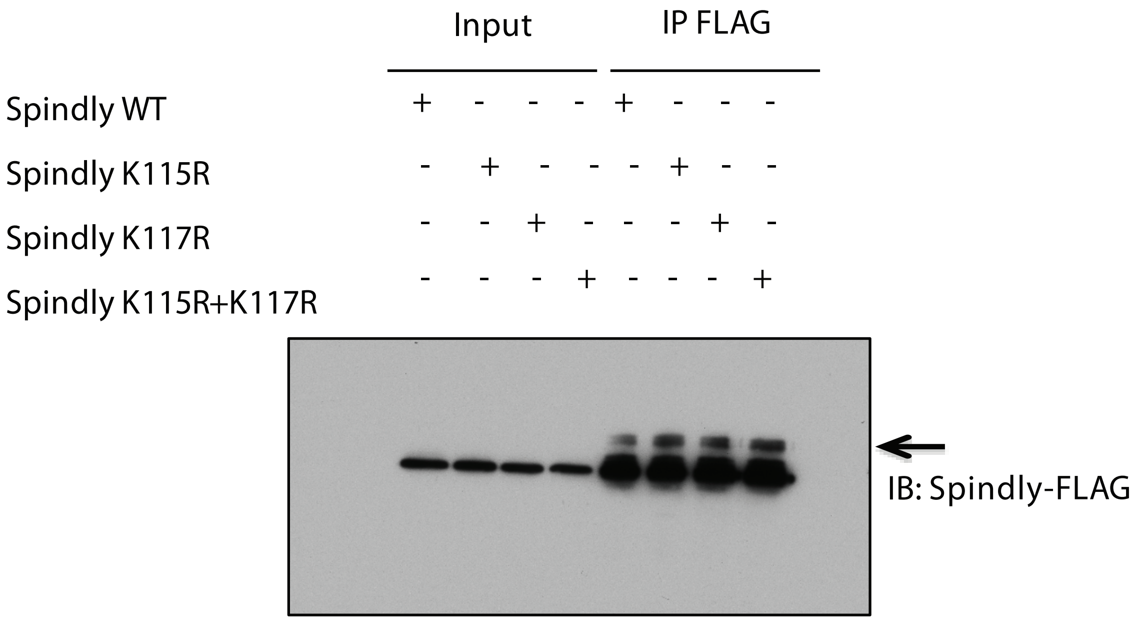
Input
IP FLAG
Spindly WT
+ - - - + - - -
Spindly K115R
- + - - - + - -
Spindly K117R
- - + - - - + -
Spindly K115R+K117R
- - - + - - - +
IB: Spindly-FLAG

A)

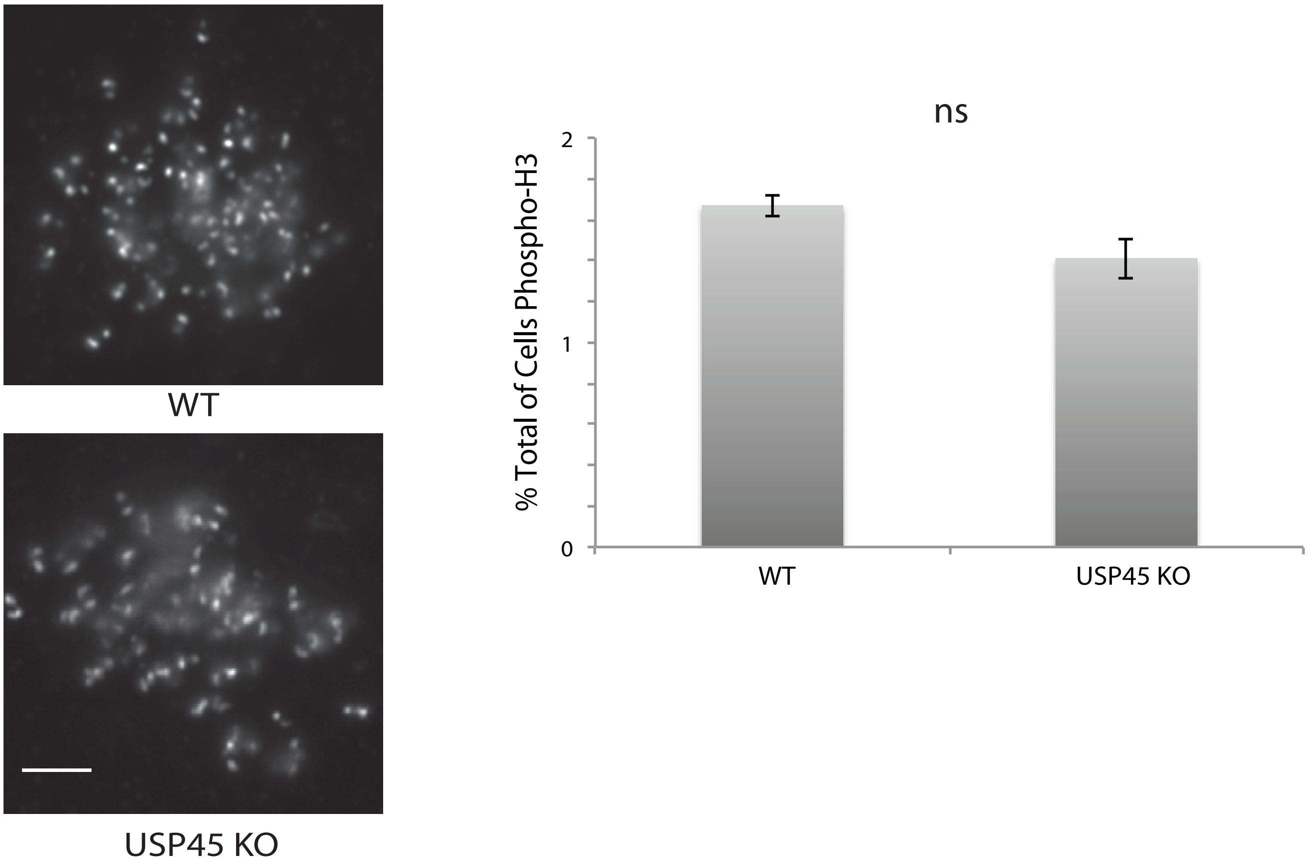

Supplement: Supplementary file 1 — Supp Info legeds and supp Figures [file 41598_2018_32685_MOESM1_ESM.pdf]
