# Supplementary material for: USP45 and Spindly are part of the same complex implicated in cell migration
Source: Sci Rep. 2018 Sep 26;8:14375. doi: 10.1038/s41598-018-32685-8 (PMC6158257; doi:10.1038/s41598-018-32685-8)

Original Blots

Figure 1B

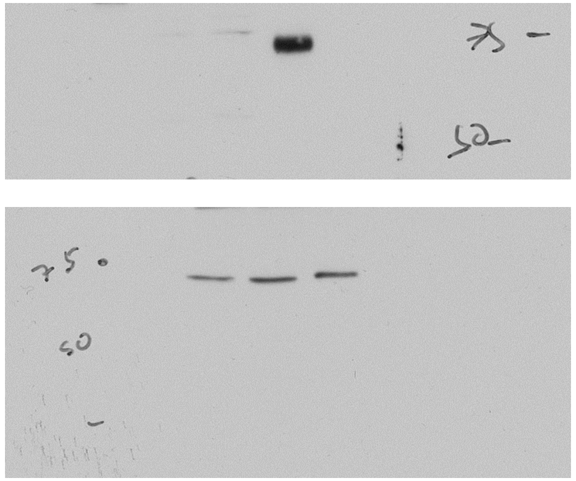

Figure 1D

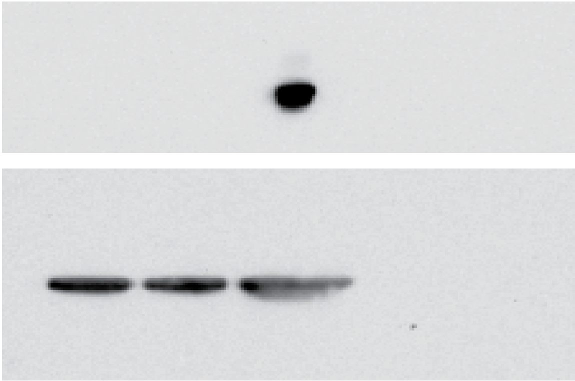

Figure 1E

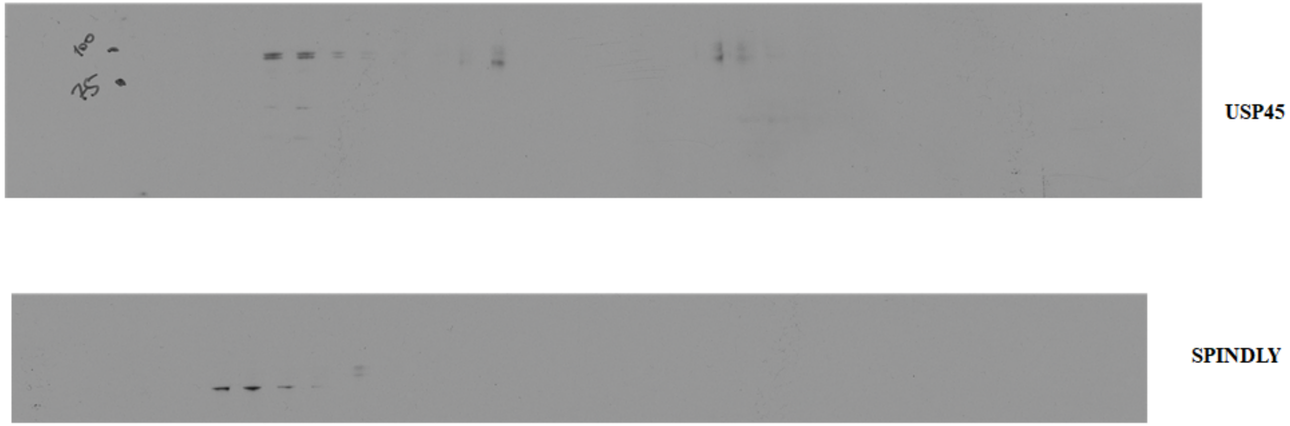

Figure 1F

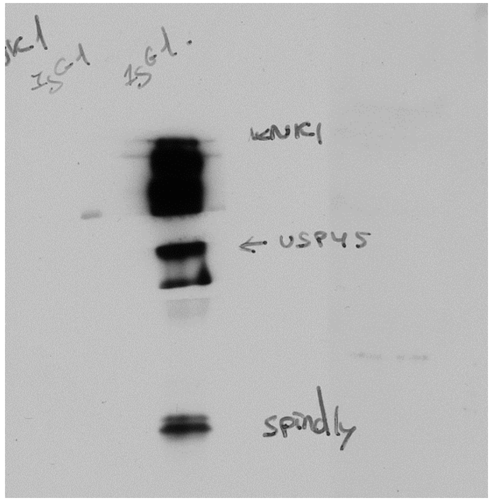

Figure 1

Figure 2A

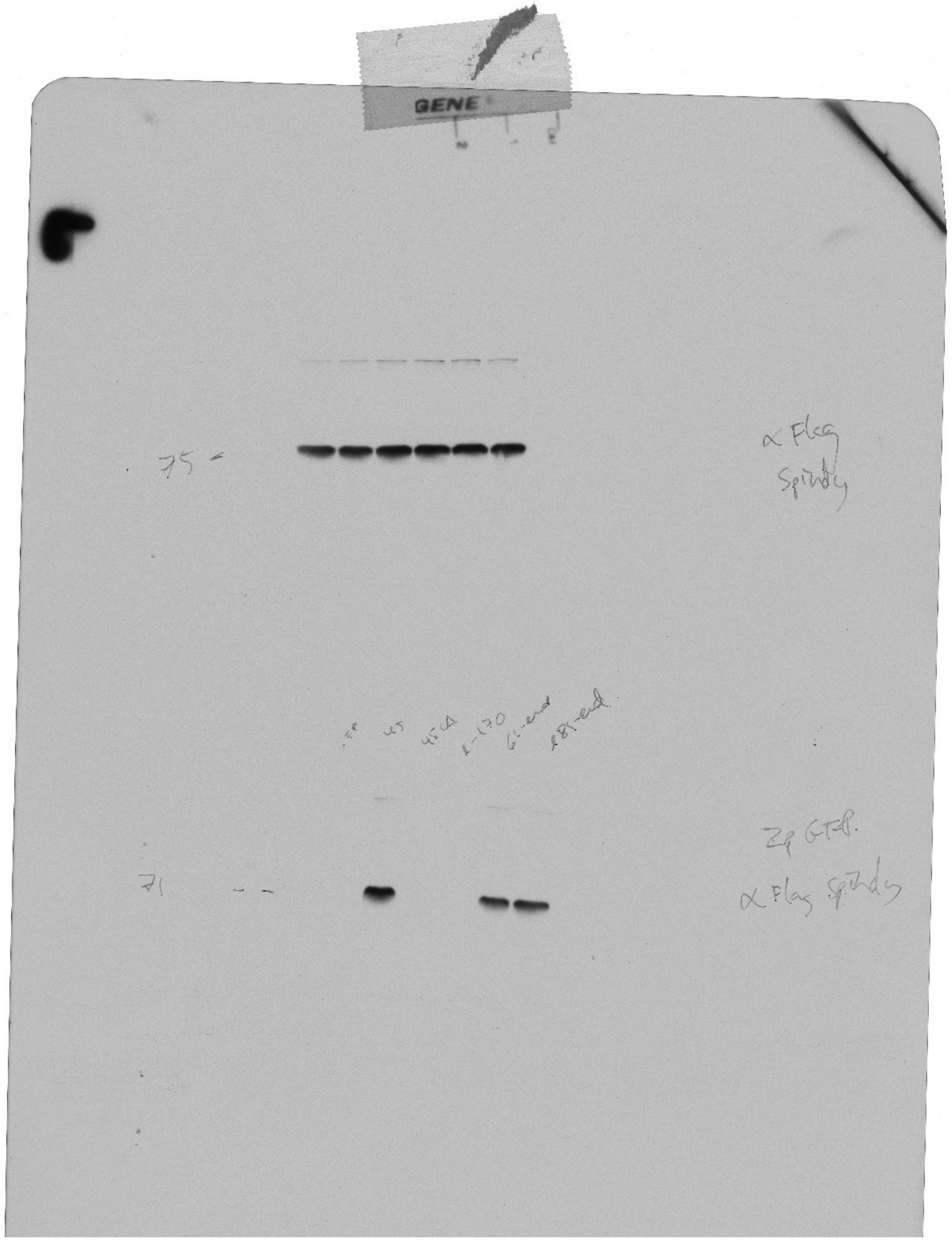

Figure 2

Figure 3A

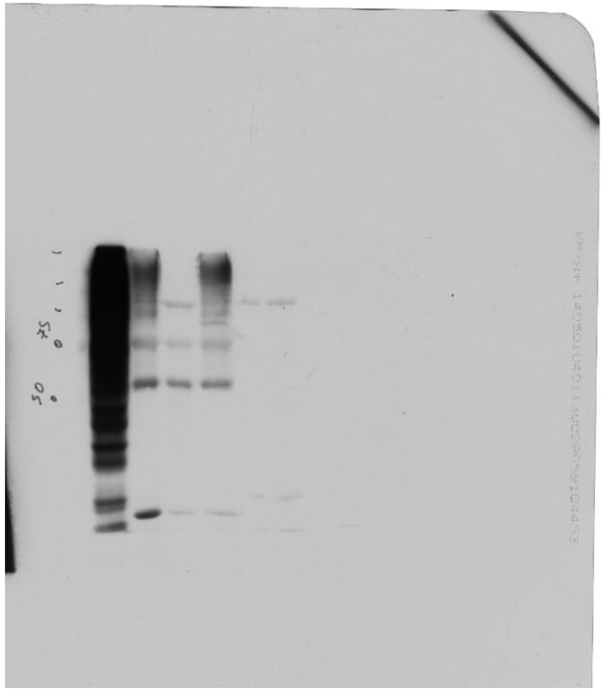

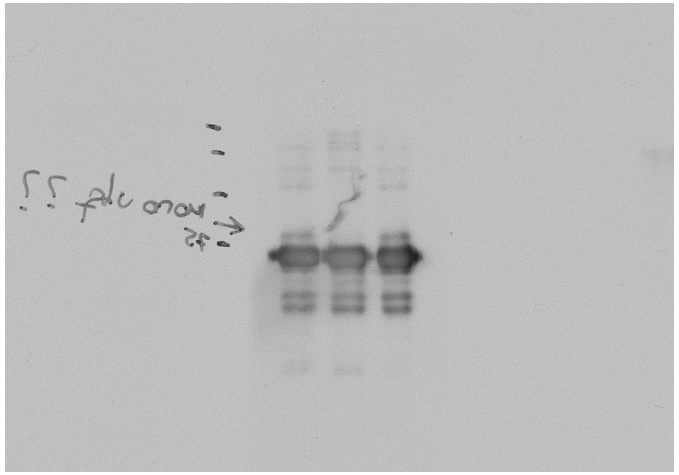

Figure 3B

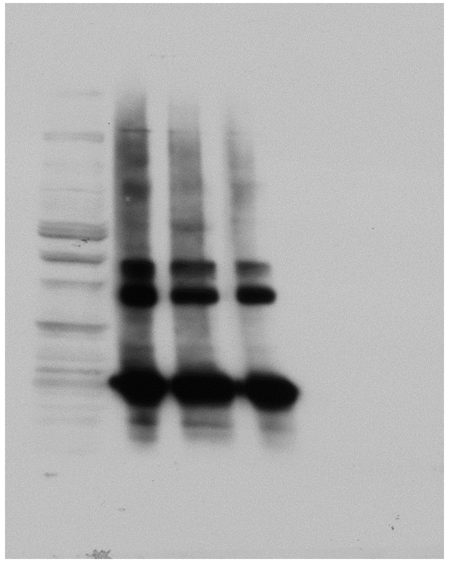

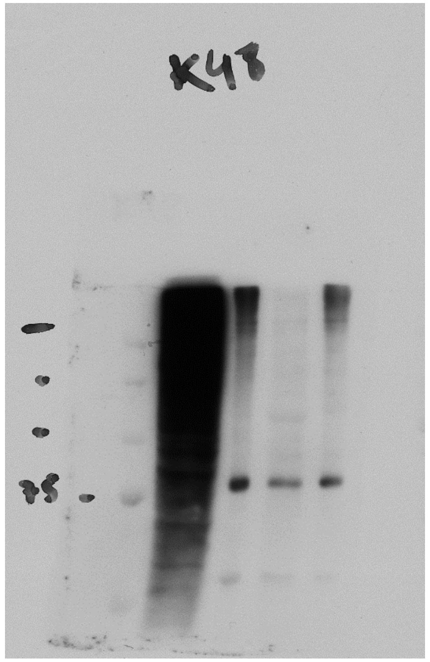

Figure 3

Figure 4A

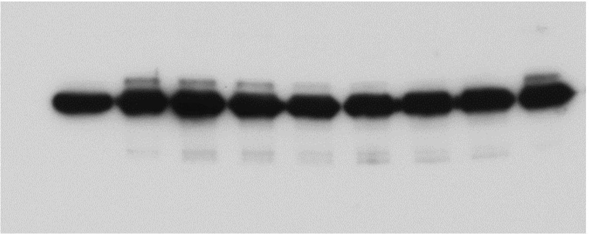

Figure 4B

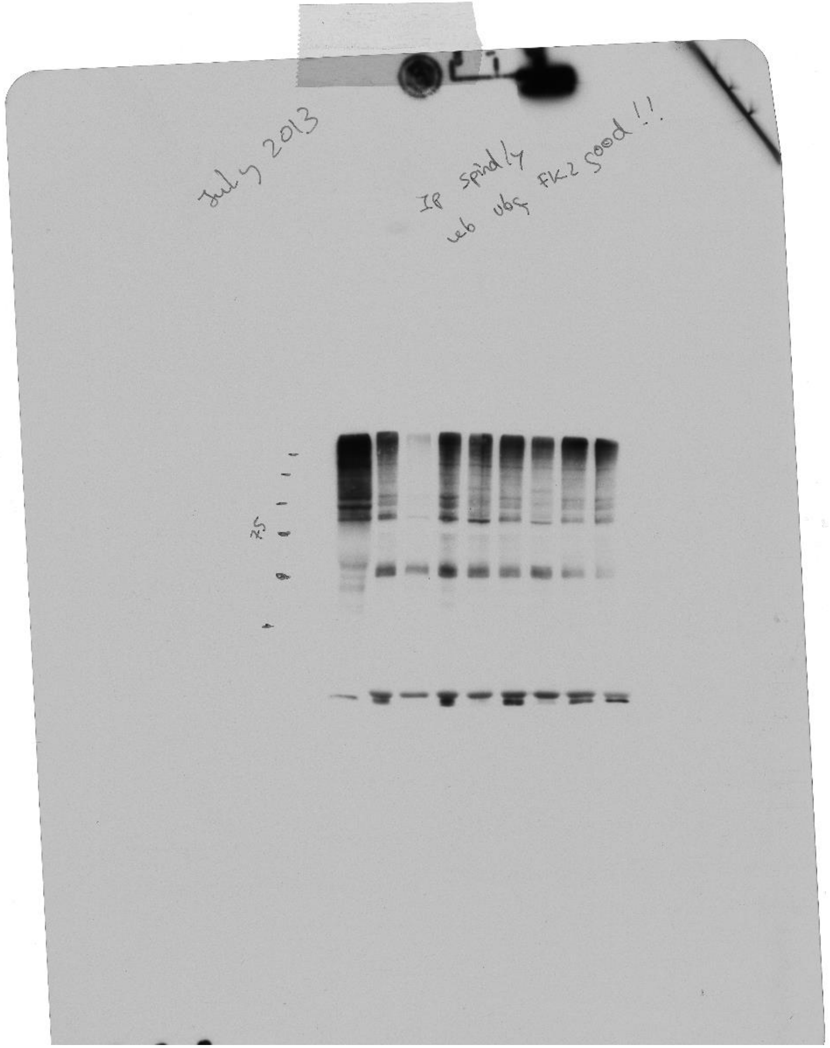

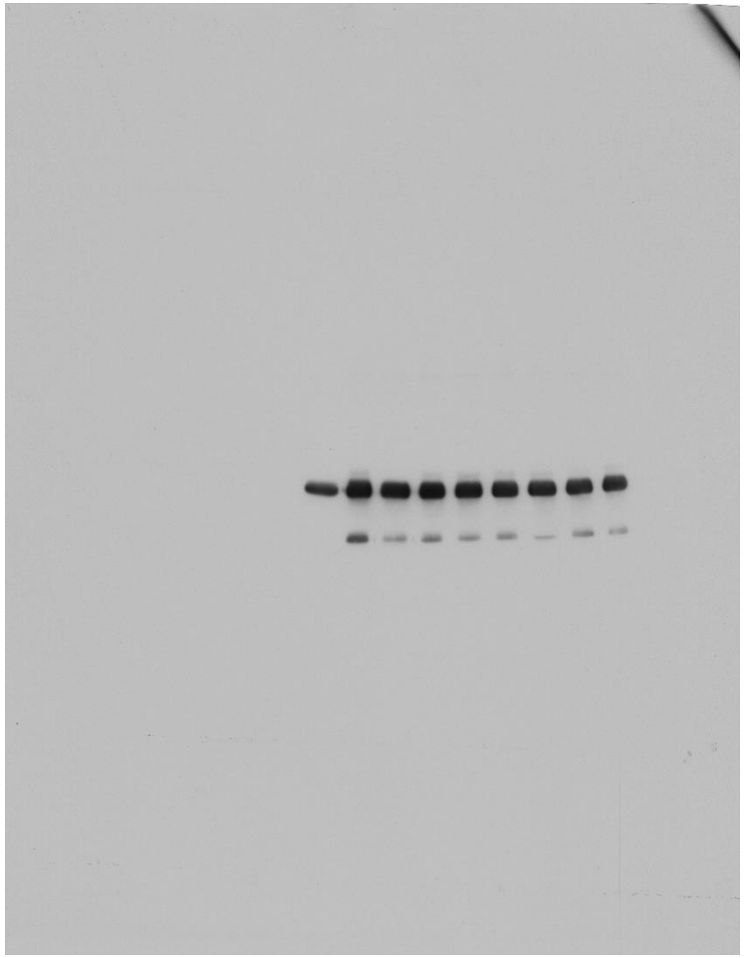

Figura 4

Supp Figure 2

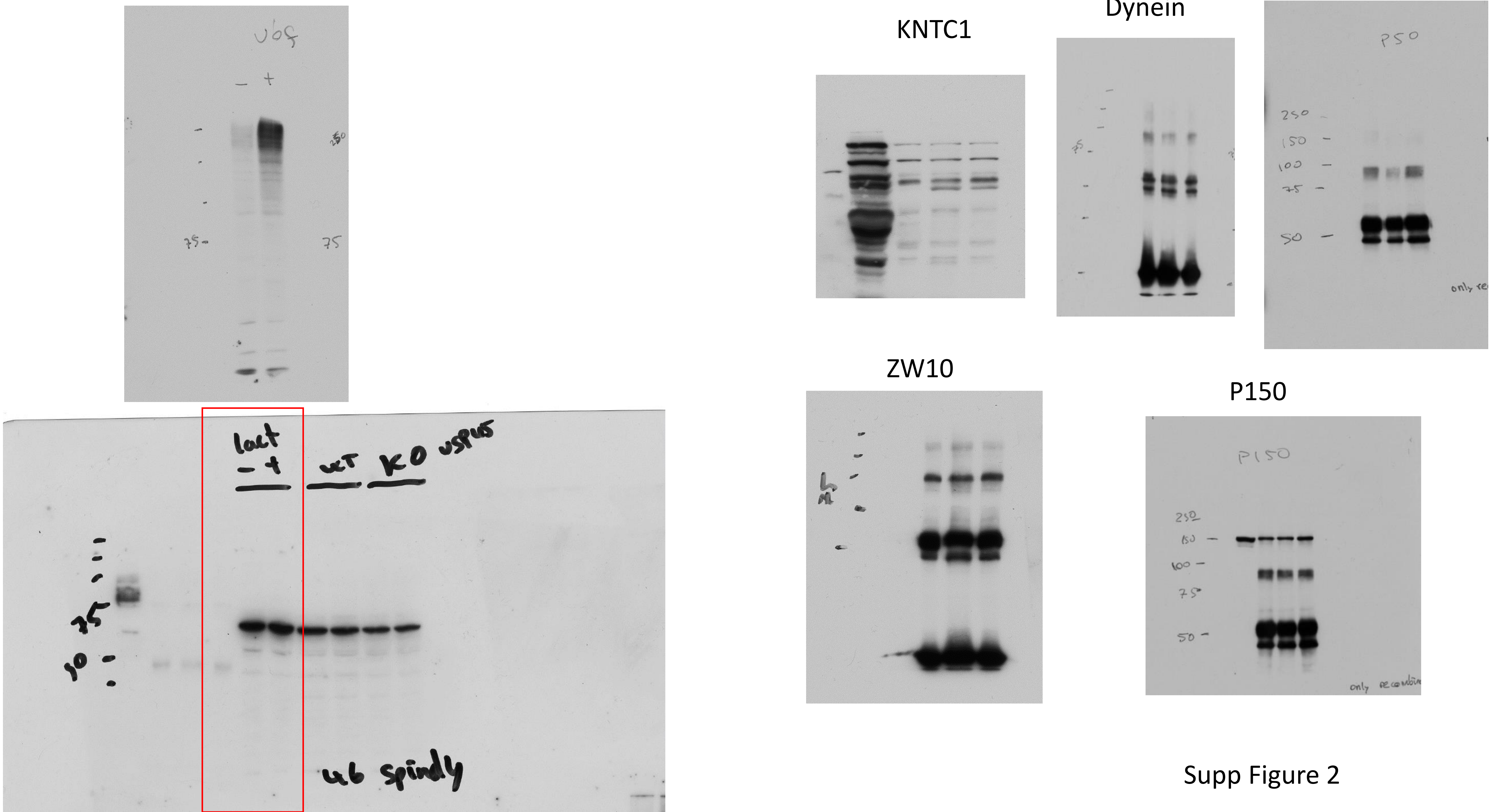

Supp Figure 2

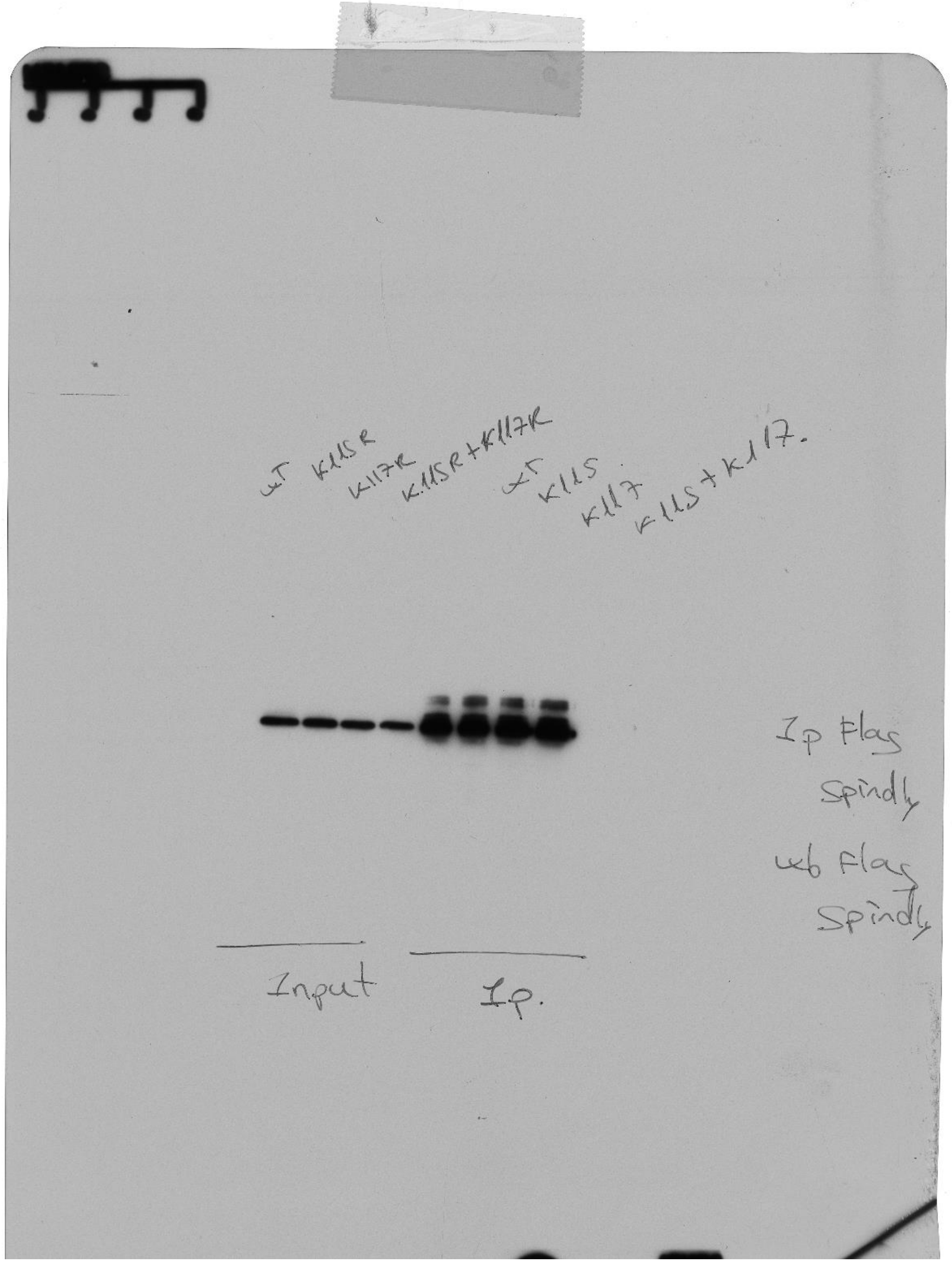

Supp Figure 3

Figure 1G

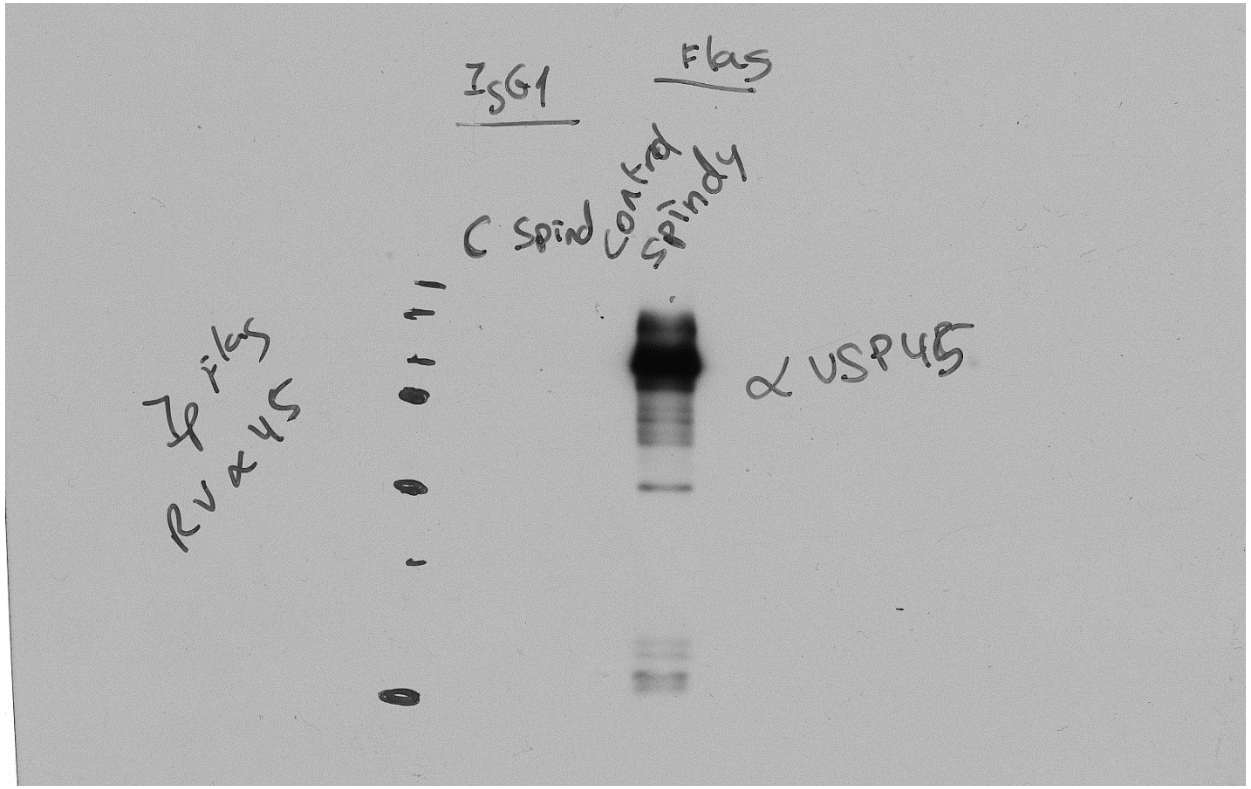
IgG1
Flag
C spind
control
spindy
α USP45

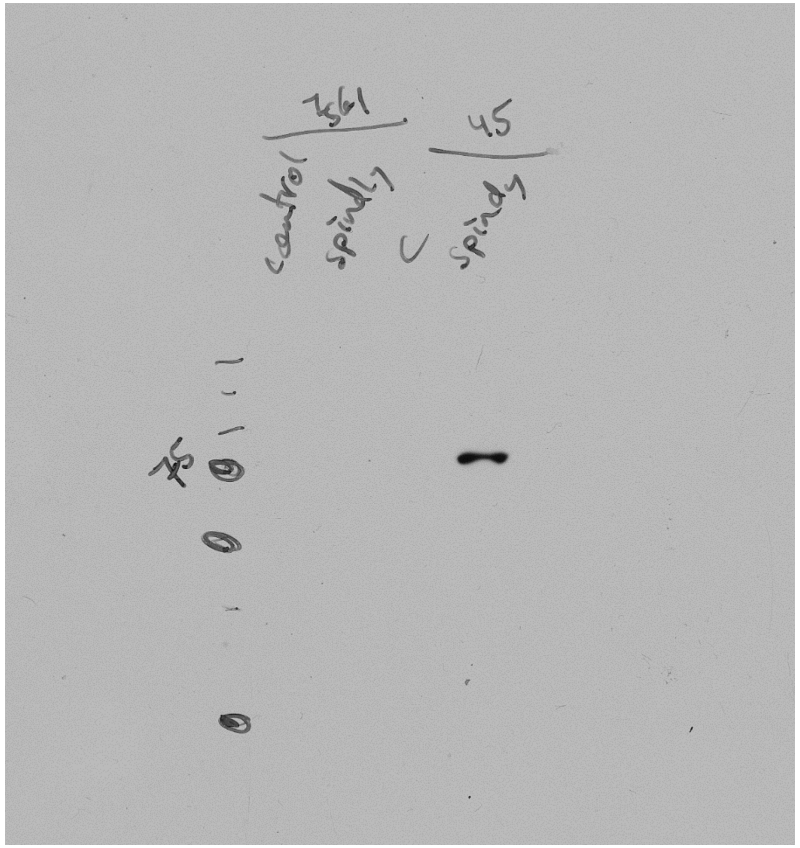
45
control
spindly
C
spindy

Part of Figure 4A

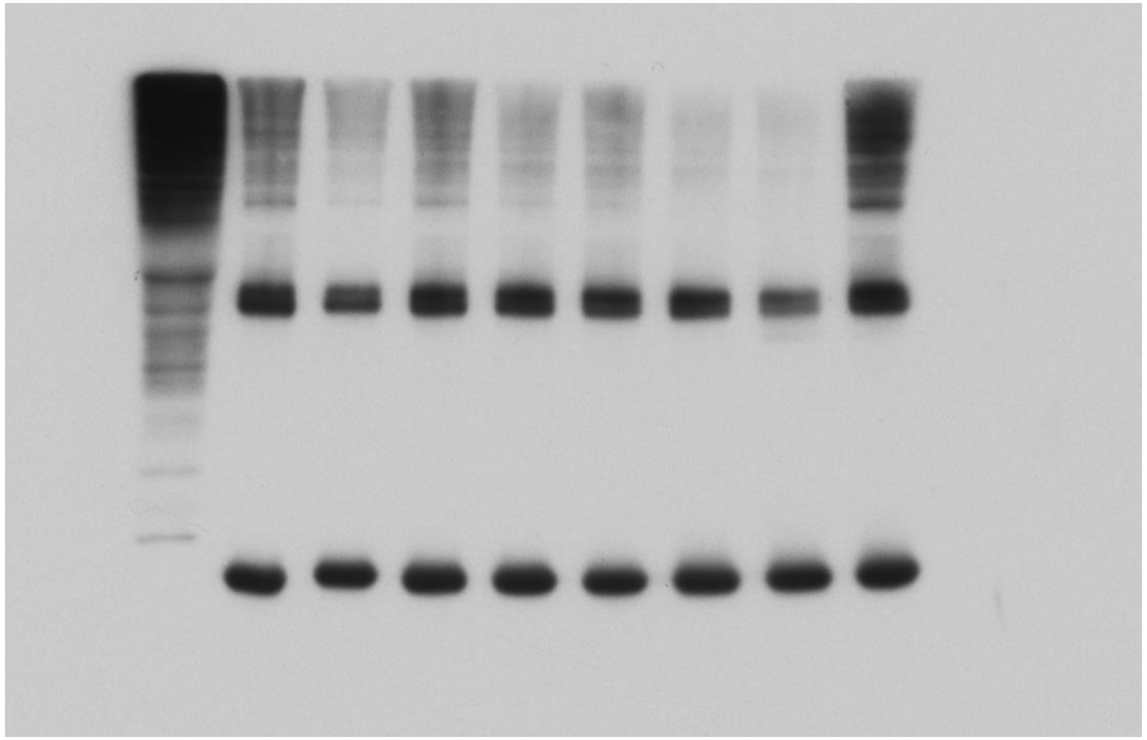

Supplement: Supplementary file 2 — Original blots [file 41598_2018_32685_MOESM2_ESM.pdf]
